# Origin of the ionic-strength dependent reentrant behavior in the liquid-liquid phase separation of uncharged intrinsically disordered proteins

**DOI:** 10.1038/s42004-026-02011-9

**Published:** 2026-04-11

**Authors:** Sayantan Mondal, Eugene Shakhnovich

**Affiliations:** Department of Chemistry and Chemical Biology, Harvard University, 12 Oxford St, Cambridge, MA USA

**Keywords:** Biophysical chemistry, Biophysical chemistry, Proteins

## Abstract

The effect of salt on coacervation of synthetic or biological polyelectrolytes and polyampholytes is well-studied. However, recent experiments showed that largely uncharged IDPs (like FUS) also undergo LLPS at physiological salt concentrations such as [C_ion_]~0.15 M, dissolve at higher salt concentration, and again phase separate at even higher salt concentrations such as [C_ion_]~3 M. Here we use analytical theory and explicit solvent coarse-grained simulations to reveal the mechanism of these transitions, which is significantly different than that of highly charged IDPs with net charge neutrality. At low [C_ion_], the ionic solution acts as a highly correlated medium conferring long-range effective attractive interactions between spatially distant monomers. In this regime, the ion concentration inside the condensate is higher than in the bulk solution. As [C_ion_] increases, the correlation length in the ionic plasma decreases, and the condensate dissolves. Second LLPS at high [C_ion_] is due to the entropy-driven crowding, and the ion concentration inside the condensate is lower than in the bulk. Our study unravels a general physical mechanism of salt-dependent reentrant behavior in LLPS in uncharged IDPs.

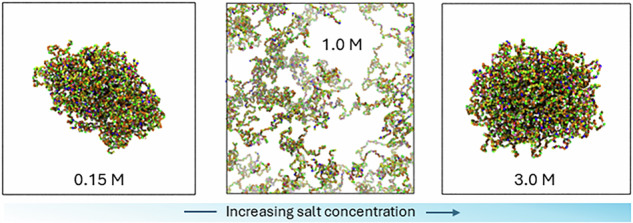

## Abstract (web summary)

Recent experiments have shown that largely uncharged intrinsically disordered proteins (IDPs) can undergo liquid-liquid phase separation (LLPS) at physiological salt concentration (~0.15 M), dissolve at increasing salt concentration (~1.0 M), and further exhibit LLPS at much higher salt concentration (~3.0 M). Here, the authors use analytical theory and explicit solvent coarse-grained molecular dynamics simulations to show that, with increasing ion concentration, condensate dissolution takes place due to a decrease in correlation length in the ionic plasma, with further LLPS at higher salt regimes resulting from entropy-driven crowding.

## Introduction

Intrinsically disordered proteins (IDPs) and genetic materials can undergo liquid-liquid phase separation (LLPS), which leads to the formation of biomolecular condensates^1^. The condensates exhibit liquid droplet-like behavior and underlie the formation of membrane-less organelles (such as nucleolus, stress granules, Cajal bodies, etc.). These organelles provide an additional means to compartmentalize subcellular processes^2^. In addition to the intracellular compartmentalization, such condensates/droplets are involved in a variety of biological processes, such as genome reorganization and transcription^3–5^, stress response^6^, noise buffering^7^, signal transduction^8^, and membrane remodeling^9,10^. Disruptions in LLPS have been connected to the onset of numerous diseases, such as neurodegenerative conditions and cancer^11^. During the past decade, there has been an increasing interest in understanding the formation, microscopic structure, and kinetics of LLPS, both experimentally and theoretically^12–19^.

Over the years, the molecular grammar of LLPS has been decoded^20,21^. Although new insights into the molecular grammar are emerging, it is well-accepted that individually weak but multivalent interactions between IDPs (such as $\pi -\pi$ stacking, cation-$\pi$, dipole-dipole, and charge-charge) are the major drivers of LLPS^22,23^. The condensation of IDPs is often favored by thermodynamics, where the enthalpic gain through the formation of several weak multivalent interactions and entropic gain from the release of bound water/counterions surpasses the conformational entropy loss of the IDP and the de-mixing entropy loss^24–26^. Although formation of a single (nearly) spherical phase is expected from a thermodynamic perspective, experiments always reveal a rather poly-dispersed multi-droplet state that has been explained theoretically from a kinetics perspective^17,19^. The droplet size distribution was shown to obey a power law in some cases^27^

The LLPS propensity of an IDP is governed by its sequence and patterning of charges, including phosphorylation sites^16,18,28,29^. Other than the sequence determinants, several physicochemical factors such as temperature, presence of co-solutes, ionic strength, pH, etc., can alter the LLPS propensity. In some cases, an interesting phenomenon called ‘*reentrant phase separation*’ is observed, where the monotonic variation of a control parameter (say, ionic strength) transforms a system from a phase-separated state to a macroscopically identical/similar phase-separated state via two distinct transitions^30^.

The RNA-binding protein FUsed in Sarcoma (FUS) undergoes LLPS via homotypic interactions. FUS is found to be enriched in the nucleus and plays several important regulatory roles. The phase diagram of FUS condensation in the temperature-concentration space is known^31^. In a recent study, Krainer et al. showed that FUS (along with four other IDPs) exhibits reentrant phase separation with respect to the salt concentration in solution^32^. FUS and other IDPs show LLPS at low (physiologically relevant) as well as at high (physiologically irrelevant) salt concentrations, but dissolve at intermediate salt concentrations. They suggested that, at a low salt concentration regime, phase separation is driven by a mixture of hydrophobic and electrostatic interactions, whereas, at a high salt concentration regime, phase separation is driven by hydrophobic as well as enhanced non-ionic interactions. While their analysis provides a microscopic insight into the dominant interactions at various solvent conditions, the physical reason behind multiple reentrant transitions in a broad range of salt concentrations remains unclear. We notice that the five protein constructs used by Krainer et al. ^32^. contain a significantly low *fraction of charged residues* (*f*_*+*_ and *f*_*-*_) and low net charge per residue (*NCPR*), especially in the disordered droplet-promoting regions. A detailed analysis based on their sequence is given in the **Supporting Information** (Table S1 and S2).

Previous studies highlighted the importance of salt in the phase behavior of charged polymers, polyelectrolytes, and polyampholytes, including IDPs^33–37^. All these studies offered an intuitive and mechanistic explanation of the effect of salt on complexation (coacervation) of charged polymers: salt/ions screen attraction between monomers of opposite charges of the charged of a polyampholyte, leading to de-mixing. In the same vein, at higher salt concentration, screening of this attractive interaction becomes more effective and a reentrant transition into the dilute phase ensues. Apparently, counterions and salt ions in polyampholytes tend to weaken or altogether prevent de-mixing, which is most effective at no salt conditions in polyampholytes. However, the salt-dependent reentrant LLPS of uncharged IDPs such as the low-complexity domains of FUS and TDP-43 may not be fully captured by the conventional electrostatic models alone^32^. Thus, the mechanism of salt-dependent LLPS of an *uncharged* IDP remains unresolved.

Here, we use explicit ion/solvent coarse-grained (CG) simulations combined with analytical statistical mechanical theory to unravel the physical origin of the reentrant LLPS in FUS and other uncharged IDPs. First, we present the observations from long-timescale coarse-grained simulations that explicitly take ions and water molecules into account and discern the essential mechanism(s) by running a series of control simulations where some factors (e.g., remaining FUS charges or salt ion charges) are abrogated one by one. Next, we present the detailed theoretical analysis based on the modified Voorn-Overbeek (VO) approximation^21^ to understand the simulation observations of FUS polymer in complex water salt solutions. The classical VO approximation has been applied to treat coacervation of polyions by replacing the short-range Flory-Huggins interaction term with electrostatic interactions between monomers of polyions in the DH approximation^21^. In variation with the classical VO approximation, here we consider short-range non-electrostatic interactions between monomers of the uncharged polymer and salt ions while treating interion interactions in the DH approximation. Statistical mechanical analysis predicts two reentrant LLPS transitions in a range of salt concentrations observed in experiments/simulations as well as the distribution of ions inside and outside of the LLPS droplet in each LLPS regime. Overall, by using these two complementary approaches, we arrive at a general explanation of the reentrant LLPS phenomenon in uncharged IDPs. Although primarily motivated by the LLPS of FUS, the framework can be extended to other IDPs with a low number of charged amino acid residues.

## Materials and Methods

The conformational space of an IDP, such as the FUS low-complexity domain (FUS-LCD, residues 1-163), is vast. Hence, molecular dynamics simulations at atomistic resolution are computationally expensive and far from probing the biologically relevant length- and timescales. Therefore, several coarse-graining strategies have emerged over the years. Here, we use the MARTINI v3.0^38^ general-purpose coarse-grained (CG) force field to model our system and correlate with the results from the analytical theory. We choose the MARTINI model due to its recent success in understanding IDP dynamics and LLPS^39–45^. In addition, MARTINI has an explicit representation of the ions and water molecules (missing in other implicit solvent CG force fields) that are essential to the present study, as discussed in the previous section. In the **Supporting Information** (Section 5), we discuss the feasibility of simulating MARTINI at high salt concentrations by comparing it with an atomistic model that predicts NaCl solubility ~6 M. Below, we outline the technical details and simulation protocols.

First, we create the structure from the sequence of human FUS-LCD, available in UniProt^46^ database (ID: P35637), by using PyMOL^47^. Then the atomistic coordinates are converted into the MARTINI CG model with the help of the *martinize2* code^48^. We randomly insert 42 copies of FUS-LCD chains inside a cubic box of dimension (30 nm)^3^. Following that, we add the required number of counterions (2 Na^+^ for each FUS chain) and excess ions (Na^+^ and Cl^-^) to maintain electrostatic neutrality as well as to maintain the desired ionic strength in the range of 0.0 M to 3.0 M. We note that one MARTINI water bead is equivalent to 4 molecules of water. We note that the experiments used FUS concentration in the order of 10 μM. At this concentration, for one FUS chain, we need an approximately (55 nm)^3^ large box, and for the condensation simulations (say, with 25 chains), we need to simulate a box as large as (160 nm)^3^. As our model has explicit ions and water beads, such large systems will be computationally expensive, especially on the timescales we are interested in. Therefore, we used a concentration of FUS, which has been previously simulated by Benayad et al. ^43^ as well as Zerze et al. ^44^. Although it is much higher than the experimental concentrations, it is not unrealistic in view of the local enhancement of IDP concentration. As a result, the phase diagram/boundaries will change, as well as the threshold value of salt concentration required for LLPS. In our study, we find that the threshold lies between 0.05 M and 0.10 M (**Supporting Information**, section 7). Therefore, the comparison with experiments is only qualitative.

As the experiments by Krainer et al. ^32^ were performed using full length construct of FUS (526 residues), we additionally simulate LLPS of full-length FUS with the same MARTINI-3 parameters used to simulate FUS-LCD. Here, we use 25 copies of full-length FUS inside a (50 nm)^3^ box filled with water and ion beads. During the simulation at three different salt concentrations, we preserve contacts in the folded regions (residues 285-371 & 422-453). We use the same simulation protocols as described in this section. The full-length FUS results agree well with the simulation observation of low-complexity FUS and are discussed in the **Supporting Information** (Fig. S6).

After a steepest descent energy minimization, we equilibrate each system for 10 ns followed by a 5 μs production run. We discard the first 2 μs from each trajectory and analyze the remaining 3 μs of the trajectory. The systems that exhibit LLPS formed a well-defined droplet within 1 μs, whereas systems with no LLPS propensity remained dispersed throughout. Here we use slightly recalibrated force field parameters by increasing the protein-water Lennard-Jones interaction strength (ε_PW_) with a scaling factor $\lambda =1.03$ that gives a reasonable transfer free-energy of FUS from the dilute to the dense phase, as also shown by Zerze et al. ^44^.

For equilibration and production run, we propagate the simulations with a timestep of 10 fs and 20 fs, respectively, using the leap-frog integrator. We use the modified Berendsen (V-rescale)^49^ thermostat (T = 298 K and τ_T_ = 1 ps^−^^1^) and the Parrinello-Rahman^50^ barostat with isotropic pressure coupling (p = 1 bar and τ_P_ = 12 ps^−1^) to maintain an NpT ensemble. For equilibration purposes, we use the Berendsen barostat^46^ with τ_P_ = 6 ps^−1^. The electrostatic interactions are screened with a dielectric constant (ε_r_) of 15 within, and van der Waals interactions are terminated at 1.1 nm with the Verlet cut-off scheme. We perform all the simulations using the GROMACS 2023.1 simulation package^51^ and conduct analyses with Plumed v2.9.0^52^. For visualizing the trajectories and creating snapshots, we use the visual molecular dynamics software (VMD 1.9.3)^53^. While finite-size effects often arise from suppressed long-wavelength fluctuations during phase transitions, MARTINI simulations mitigate these issues by utilizing short-range interaction cutoffs and reaction fields. Given that our box dimensions (30–40 nm) are significantly larger than both the 1.1 nm cutoff and individual chain dimensions, as well as the condensate dimensions (Fig. S10), we do not expect significant finite-size artifacts.

For the potential of mean force (PMF) calculations between two FUS monomers, we use well-tempered metadymaics^54^, WT-MetaD (T = 300 K and BiasFactor = 5.0) with the inter center-of-mass distance (r_COM_) as the collective variable. We solvate two FUS chains in a 30 nm cubic box with MARTINI water and ions. We set the MetaD hill width to be 0.35 nm and the initial hill height to be 1.0 kJ/mol. The MetaD hills are deposited every 1 ps, and the MetaD simulations are run for 5 μs. We use PLUMED (v 2.9.0)^52^ patched with GROMACS^51^, to perform the MetaD simulations and subsequent analyses.

To quantify the extent of LLPS, we calculate a contact order parameter (COP), $Q(t)$ as shown in Eq. (1). The COP at a particular timeframe, *t* is expressed as:

1 $$Q(t) = 	{\sum}_{i,j}{q}_{ij}(t),and\\ {q}_{ij}(t) = 	\frac{1-{({r}_{ij}(t)/{r}_{0})}^{6}}{1-{({r}_{ij}(t)/{r}_{0})}^{12}}$$

where ${r}_{{ij}}\left(t\right)$ is the distance between the i^th^ and j^th^ beads at time *t* and *r*_*0*_ is fixed to be 0.5 nm. Hence, ${q}_{{ij}}\left(t\right)$ can vary smoothly from 1 to 0 for a given pair. Unlike discrete cutoff methods that utilize a Heaviside step function, Eq. (1) employs a continuous switching function to calculate contact orders. This approach provides a differentiable parameter that is more robust to small conformational fluctuations and better captures the distance-dependent nature of residue interactions.

We further define $\Delta Q$ as the gain in COP by subtracting the time-averaged COP for FUS with only the counterion system, ${Q}_{0}$. Therefore, $\Delta Q=\left\langle Q(t)\right\rangle -{Q}_{0}$. The pair-interaction energies are obtained using ‘*gmx energy*’ code. The energies contain both Lennard-Jones and electrostatic interactions.

## Results

**Fig. 1:**
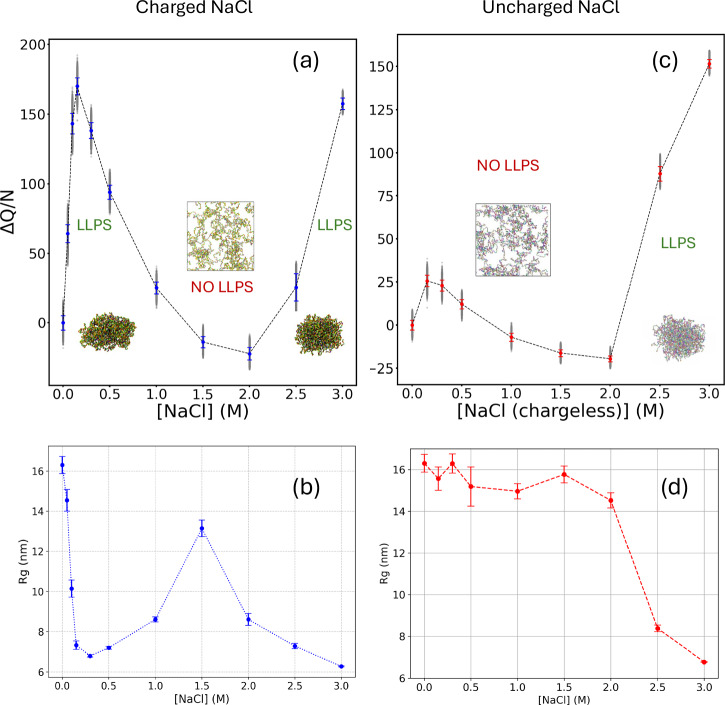
Reentrant LLPS of FUS-LCD with charged and uncharged ions. Time-averaged contact order parameter (ΔQ) per FUS-LCD chain against the ionic strength of the solution: **a** when sodium and chloride ions retain their charges, and **c** when the sodium and chloride ions are made chargeless LJ beads. An increase in $\varDelta Q/N$ above a threshold indicates condensation. **Time averaged radius of gyration (for all IDP chains in the box), Rg against the ionic strength of the solution:** **b** when sodium and chloride ions retain their charges and **d** when the sodium and chloride ions are made chargeless LJ beads. A decrease in Rg indicates condensation. Both **a** and **b** show the ‘double reentrant’ phenomena. The dotted lines should be used as guides to the eye.

First, we study the propensity of FUS to undergo LLPS in the range of ion concentrations from 0.0 M to 3.0 M as observed in experiment^32^. We observe no condensation at 0 M. Starting from physiological 0.10 M salt, FUS undergoes LLPS into a single compact cluster, which dissolves beyond 1.0 M NaCl. Interestingly, LLPS reappears when salt concentration is above 2.5 M (Fig. 1a). This ‘extremely high’ salt concentration regime, although biologically non-relevant, is important to understand from a physics perspective as noted below.

In Fig. 1a, we plot the density of FUS $\Delta Q/N$ against salt concentration, [NaCl]. A sharp increase can be observed from [NaCl]=0.0 M to 0.15 M. The value of $\Delta Q/N$ decreases upon further increase of salt concentration, and the LLPS cluster fully dissolves when [NaCl] exceeds 1.0 M. However, when [NaCl] > 2.0 M, the value of $\Delta Q/N$ increases again and reaches the values observed at 0.15 M [NaCl]. This is the second entrance into the ‘LLPS’ regime from the ‘no LLPS’ zone. Therefore, we observe the double reentrant transition, where the second reentrance is observed as in experiments^32^. From the error bars (or standard deviations) Fig. 1a, important information regarding the dynamics of the condensates can be gleaned. Although condensation/LLPS occurs at around [NaCl]=0.15 M and at around [NaCl] = 3.0 M, the condensate is more dynamic for the [NaCl] = 0.15 M system (more dispersed data), and at [NaCl] = 3.0 M, the condensate is less fluctuating (less dispersed data). To monitor the transition from a dispersed to a condensed state, we additionally utilized the global radius of gyration (Rg) of all chains within the simulation volume as a collective order parameter. It is important to distinguish this system-wide measure from the single-chain Rg typically discussed in polymer scaling theories^55–57^. In our results, a decrease in global Rg signifies the formation of a compact multi-chain condensate. As shown in Fig. 1b, Rg sharply decreases for [NaCl]~0.00M–0.50 M followed by an increase around [NaCl]~1.0 M and again decreases at higher [NaCl].

In Fig. 1c we plot the same quantity as in Fig. 1a (that is, $\Delta Q/N$ vs [NaCl]), however, for an artificial control system with uncharged NaCl (essentially LJ beads). This is equivalent to ‘turning off’ the electrostatic interactions in the analytical approach below. There is a slight increase in $\Delta Q/N$ at lower salt concentrations (probably due to depletion forces), but without condensation. The absence of condensation can be understood from the high Rg values at that salt concentration (Fig. 1d) and from visual inspections. Interestingly, FUS undergoes LLPS at high salt (here, LJ bead) concentration, indicated by a sharp increase in $\Delta Q/N$ (Fig. 1c) and a decrease in Rg (Fig. 1d). The analytical theory, discussed later in the manuscript, also predicted that the system would undergo phase separation at high salt concentrations when electrostatic interactions are ‘turned off’. Plots for pairwise interaction energies against salt concentrations, namely ${E}_{{FUS}}$, ${E}_{{FUS}-{Ion}}$, ${E}_{{FUS}-W}$, and ${E}_{{Ion}}$ can be found in **Supporting Information** (Figs. S2 and S3). We additionally provide the single-chain end-to-end distance variation with [NaCl] in Fig. S12.

**Fig. 2:**
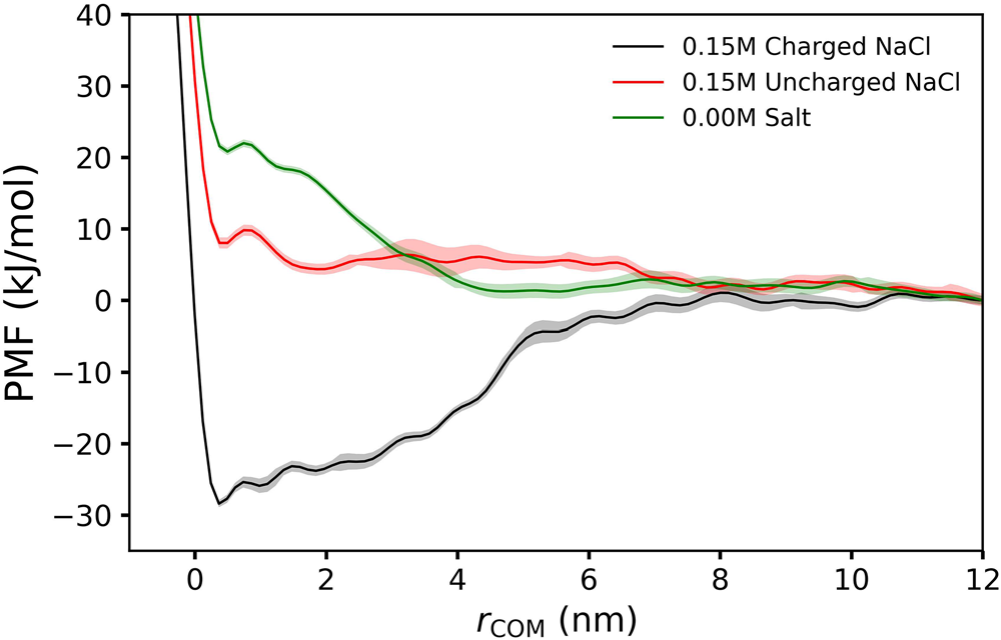
Effective attraction between FUS-LCD monomers through a highly correlated medium of salt ions. Potential of mean force (PMF) between two FUS chains calculated by using well-tempered metadynamics. The two FUS chains are attractive in 0.15 M NaCl solution but become repulsive when the ionic charges are set to ‘0’, that is, electrostatics are turned off; or when no excess ions are present at 0.0 M.

From the well-tempered metadynamics (WT-MetaD) calculations, we obtain the potential of mean force (PMF) between two FUS-LCD chains in 0.15 M NaCl solution, with and without the ionic charges. The resultant PMFs are shown in Fig. 2 where the blue trace shows an effective attraction between two FUS chains when electrostatics are ‘on’, with approximately 30 kJ/mol free energy stabilization in the associated state. In the same figure, the red and green traces show an effective repulsion between the FUS chains when electrostatics are either ‘off’ or at [NaCl] = 0.0 M. This demonstrates the long-range (~10 nm) effective attraction through the ionic media, which is absent in the presence of inert crowders or no salt ions.

Next, we explore in detail the mechanism of LLPS of FUS, including crucially, the role of ions. For control and comparison, we study several model systems with the same box size and number of FUS molecules. The five systems are as follows: (1) Original model of FUS at 0.15 M NaCl, (2) artificial ‘chargeless’ FUS chains in 0.15 M NaCl where a few charges of FUS chains are abrogated, (3) FUS with artificial ‘chargeless’ 0.15 M NaCl whereby charges of salt ions are set to zero, (4) FUS with only a few Na^+^ ions serving as counterions to FUS charges, and (5) FUS with only 0.15 M Na^+^ ions without any Cl^-^ ions. The last system contains a high amount of net positive charge. Below, we detail the rationale behind studying these systems.

**Fig. 3:**
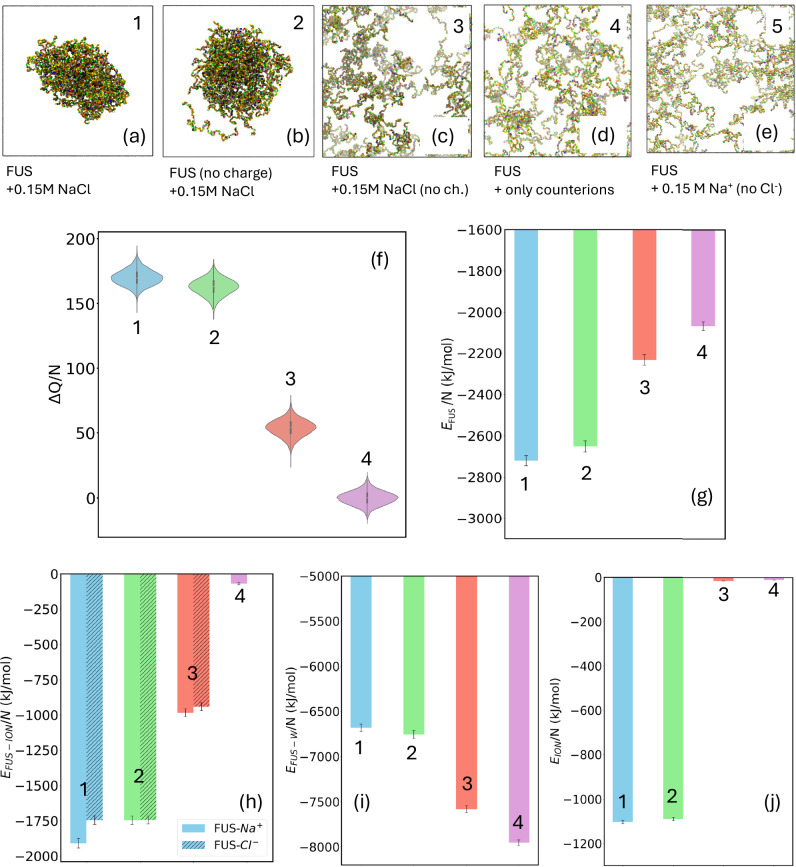
Representative snapshots, number of contacts, and pair-interaction energetics from coarse-grained molecular dynamics. **a** FUS chains in 0.15 M NaCl shows condensate formation, **b** Artificial FUS chains with no charges in in 0.15 M NaCl shows condensate formation, **c** FUS chains in 0.15 M artificial NaCl with no charges (which behave like LJ beads) shows no condensate formation, **d** FUS with only Na^+^ (2 ions per chain to maintain electrostatic neutrality) ions shows no condensate formation, and **e** FUS with 0.15 M excess Na^+^ (no Cl^-^) ions shows no condensate formation. The first four systems are respectively denoted as 1, 2, 3, and 4 in the subsequent plots. **f** Variation of the contact order parameter ($\varDelta Q$) per FUS chain for the first four systems. Time-averaged interaction energies of different pairs: **g** Among FUS chains, **h** Between FUS and ions, **i** Between FUS and water beads, and **j** Among the ions.

The purpose of the control system-2 is to check whether the 4 charges on FUS (N-ter, C-ter, Asp-5, and Asp-46) drive LLPS in any way. It turns out that FUS, whose own charges are set to 0, forms condensate in the regular NaCl solution whose salt ion charges are intact (Fig. 3b). We confirmed these observations for system-2 by starting from a completely dispersed initial state as well (**Supporting Information**, Fig. S4). Therefore, we can rule out the role of FUS counterion-mediated inter-polymeric interaction in the FUS LLPS. The purpose of system-3 is to test whether the ionic charges are required for LLPS in the low [NaCl] regime as predicted by the analytical theory (see below). In agreement with the theoretical prediction, the condensate does not form in this case (Fig. 3c). The purpose of the control system 4 (Fig. 3d) is to demonstrate that FUS, with only its counterions (that is, 2 Na^+^ ions per chain), cannot exhibit LLPS. Importantly, in control system 5 (Fig. 3e), where Na^+^ ions retained their positive charges and the Cl anions were absent, the highly correlated Debye-Huckel plasma did not exist, and FUS did not form a condensate. This supports the conclusion from the analytical theory (discussed below) that LLPS at low salt concentration is driven by the interactions through highly correlated ionic plasma. All in all, we conclude that ha ighly correlated system of oppositely charged ions, a Debye-Huckel plasma, is driving reentrant condensation and dissolution of FUS at low [NaCl]. This mechanism has significant similarity to polymer-salt-induced condensation of DNA or psi($\psi$)-condensation^58,59^, discussed subsequently.

To quantify the extent of LLPS, in Fig. 3f we plot $\Delta Q$ normalized by the number of FUS chains for the four systems depicted in Fig. 3a through Fig. 3d. For systems 1 and 2, the gain in the number of contact pairs is at the maximum. On the contrary, for systems 3 and 4, the value of $\Delta Q/N$ is much lower. Note that these are not the absolute values mentioned in the **Methods** section. In Fig. 3g we show the time-averaged pair interaction energies (LJ + Coulomb) between FUS chains, ${E}_{{FUS}}/N$. As one can presume, the interaction between FUS chains is the strongest for system 1, and almost comparable for system 2. However, these energies are substantially lower for systems 3 and 4, where the interaction primarily arises due to intra-chain contacts. In Fig. 3h, the energies of FUS-ion interaction are plotted, separately for Na^+^ and Cl^-^. The values for systems 1 and 2 are lower, which indicates enhanced interaction of FUS with salt ions. The FUS chains of system 1 interact slightly stronger with the Na^+^ than with the Cl^-^ owing to the presence of two negatively charged aspartate residues in each chain. Such a distinction is not present in system 2, where FUS is made chargeless. In system 3, the excess ions are chargeless, but the counterions (2 Na^+^ per chain) are not. Hence, it shows enhanced interaction with Na^+^, but the values are almost 50% reduced. System-4 has no Cl^-^ and only Na^+^ counterions. A reverse trend is observed in Fig. 3i where the energy of FUS with water is plotted. Systems 1 and 2 interact less with water compared to systems 3 and 4. Figure 3j shows the interaction energies of ions. Here we find that ions interact favorably when LLPS occurs. Overall, from the pair-energetic,s we find that LLPS of FUS stabilizes the inter-FUS, FUS-ion, and inter-ionic interactions; and destabilizes the FUS-water interactions.

**Fig. 4:**
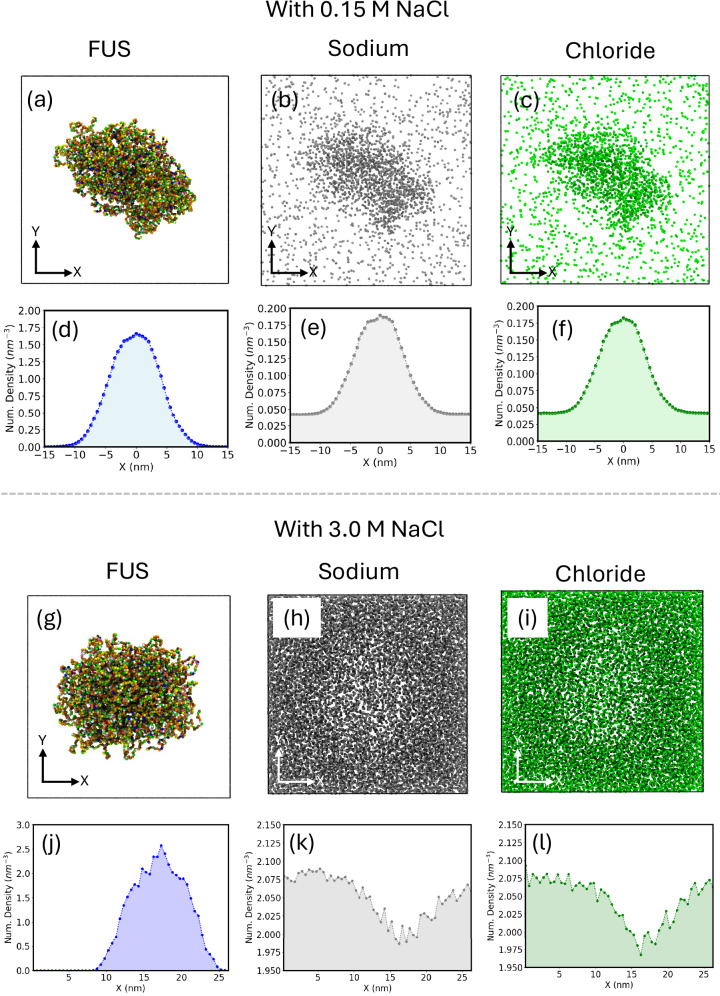
Spatial distribution of the FUS condensate, sodium, and chloride ions inside the simulation box for two different ionic strengths, namely, 0.15 M and 3.0 M. In the low ion concentration regime, the density of ions follows the density of FUS as shown pictorially in (**a**)–(**c**); and quantitatively from the spatial density profiles in (**d**)–(**f**). On the contrary, in the high ionic concentration regime, the FUS condensate expels ions from its interior as shown pictorially in (**g**)–(**i**); and quantitatively from the spatial density profiles in (**j**)–(**l**).

**Fig. 5:**
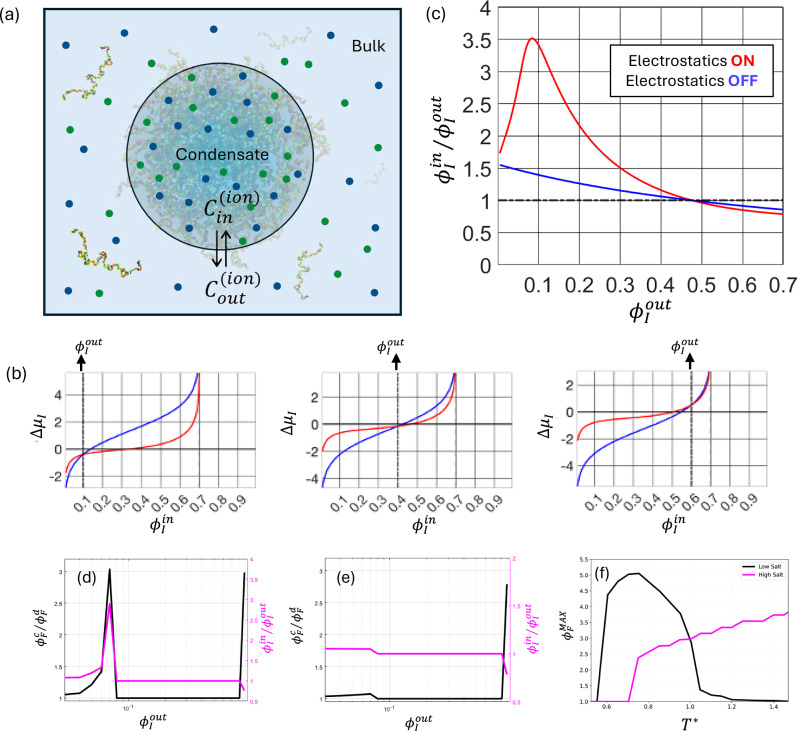
Understanding LLPS of uncharged IDPs from statistical mechanics-based analytical theory. **a** A schematic illustration of the liquid-liquid phase-separated system. The IDP rich phase is the condensate with volume fraction of ions ${\phi }_{I}^{in}$ and the dilute/bulk phase with volume fraction ${\phi }_{I}^{out}$. **b** Graphic solution of Eq.(3) for the difference of chemical potential of ions in the condensed phase vs. the bulk as a function of volume fraction of salt inside the condensate (${\phi }_{I}^{{in}}$). Three panels correspond to different values of ${\phi }_{I}^{{out}}$ as marked by vertical dashed lines (0.1, 0.4, and 0.6, respectively). Red lines correspond to the full free energy functional with electrostatics between ions, while the blue lines are a control where charges are “turned off”, i.e.,. A = 0. The values of ${\phi }_{I}^{in}$ at which the lines intersect y = 0 correspond to the solutions of Eq. (3) for ${\phi }_{I}^{in}$. **c** Relative volume fraction of salt inside the condensate vs that in the dilute phase representing solution of Eq. (5) in the full range of volume fractions of salt. **d** Reentrant transitions in the solution of FUS with ${\widetilde{\chi }}_{{FI}}=-2{kT}$. At low salt concentration, FUS first condenses, and as salt concentration increases, condensates dissolve back into a one-phase solution. Enrichment of salt inside the condensate drives the first transition until the second phase separation transition, accompanied by depletion of salt in the condensate. The Black curve shows the volume fraction of FUS in the condensate relative to the dilute phase. The Magenta curve shows the volume fraction of ions in the condensate relative to the dilute phase. **e** same as **d** but with ion charges off (A = 0). **f** Temperature dependence of the volume fraction in the FUS condensate in the low salt phase (black lines) and the high salt phase (magenta).

Next, we study the spatial distribution of ions. Observations from our CG simulations are presented in Fig. 4. We find that, in LLPS occurring at low salt concentration, the condensate will absorb more ions, and in the second LLPS occurring at higher concentration, the condensate will expel ions. The analytical theory presented below, also predicts the same (Fig. 5b–d). For the condensate at [NaCl]=0.15 M, there is a significantly increased population of both Na^+^ and Cl^-^ ions in the interior of the FUS condensate, as seen from the representative simulation snapshots (Fig. 4a–c). In Fig. 4d, Fig. 4e, and Fig. 4f we respectively plot the spatial density profile of FUS, sodium, and chloride along the X-dimension of the simulation box. The density profile clearly shows the excessive adsorption of ions by the FUS condensates. In the case of full-length TDP-43, Ingolfsson et al. foundsimilar increased ionic concentration inside the condensate, from MARTINI-3 CG simulations^60^.

In the high salt concentration LLPS regime, for [NaCl] = 3.0 M, the opposite effect is observe,d where the FUS-rich region of the box has a lower density of ions than the region outside the condensate. It can be seen in the snapshots (Fig. 4g–i) and also from the density profile plots (Fig. 4j–l). This shows moderate expulsion of ions from the interior after reentrance. Also note that excessive adsorption of ions in the low salt regime is much stronger than their expulsion in the high salt regime (almost 3-fold for adsorption and ~15% for expulsion), again in agreement with the predictions of the analytical theory as highlighted in Fig. 5 below.

### Theoretical analysis

In this section, we develop a simple mean-field theory to get a deeper insight into the surprising findings from simulations that point to the crucial role of NaCl ions in *promoting* LLPS at low ionic strengths. These findings are somewhat surprising as it contradicts common intuition borne out of studies of net neutral polyampholytes and coacervating polyelectrolytes, where salt ions weaken LLPS by screening the attraction between oppositely charged residues^61^. The detailed steps and descriptions of the analytical theory can be found in the **Supporting Information** (Section 1).

Consider the phase-separated solution of an uncharged IDP (for example, FUS-LCD), which we model as an uncharged homopolymer. The dense phase occupies a volume $V$ (together with water and ions), and the remaining volume $\left({V}_{0}-V\right)$ is occupied by the dilute phase. We use the Voorn−Overbeek (VO)^34^ approximation to describe the thermodynamics of an uncharged polymer in the Flory-Huggins approximation and charged salt ions in the Debye-Huckel approximation. In addition to the usual electrostatic interactions in the VO theory, we included direct *non-electrostatic* LJ interactions between uncharged FUS and salt ions. The total free energy of the system is given by:

2 $$\begin{array}{l}{F}_{total}=V\left[\begin{array}{l}\frac{1}{L}T({\phi }_{F}^{in}){{{\mathrm{ln}}}}({\phi }_{F}^{in})+T{\phi }_{I}^{in}\,{{{\mathrm{ln}}}}({\phi }_{I}^{in})+T(1-{\phi }_{F}^{in}-{\phi }_{I}^{in}){{{\mathrm{ln}}}}(1-{\phi }_{F}^{in}-{\phi }_{I}^{in})+\\ +{\tilde{\chi }}_{FF}{({\phi }_{F}^{in})}^{2}+{\tilde{\chi }}_{FI}{\phi }_{F}^{in}{\phi }_{I}^{in}+{F}_{DH}({\phi }_{I}^{in})\hfill\end{array}\right]+\\ +({V}_{0}-V)\left[\begin{array}{l}\frac{1}{L}T({\phi }_{F}^{out}){{{\mathrm{ln}}}}({\phi }_{F}^{out})+T{\phi }_{I}^{out}\,{{{\mathrm{ln}}}}({\phi }_{I}^{out})+T(1-{\phi }_{F}^{out}-{\phi }_{I}^{in}){{{\mathrm{ln}}}}(1-{\phi }_{F}^{out}-{\phi }_{I}^{out})+\\ +{\tilde{\chi }}_{FF}{({\phi }_{F}^{out})}^{2}+{\tilde{\chi }}_{FI}{\phi }_{F}^{out}{\phi }_{I}^{out}+{F}_{DH}({\phi }_{I}^{out})\hfill\end{array}\right]\end{array}$$

Where $\phi$ denotes volume fraction of ions and FUS inside the condensate or outside as indicated by corresponding subscripts/superscripts (see Eq. S1 for definition of volume fractions). Square brackets denote the free energy of FUS and ions in and out of the condensate. Flory-Huggins parameters $\tilde{\chi }$ describe effective *non-electrostatic* LJ interactions between FUS, and the ions, where explicit water is excluded via the incompressibility conditions given in Eq. S1.

We assume, for simplicity, that salt anions and cations have equal non-electrostatic interactions with both water and FUS and do not make a distinction between them. Electrostatic interactions are between salt ions only and they are described by the free energy contribution ${F}_{DH}$ in the Debye-Huckel approximation as detailed below.

The ion-equilibrium between the condensate and the bulk is achieved at equal chemical potentials of ions in the condensate (“in”) and the bulk (“out”):

3 $$T\,{{{\mathrm{ln}}}}\left(\frac{{\phi }_{I}^{in}}{{\phi }_{I}^{out}}\right)-T\,{{{\mathrm{ln}}}}\left(\frac{1-{\phi }_{I}^{in}-{\phi }_{F}^{in}}{1-{\phi }_{I}^{out}-{\phi }_{F}^{out}}\right)+{\mu }_{DH}({\phi }_{I}^{in})-{\mu }_{DH}({\phi }_{I}^{out})+{\tilde{\chi }}_{Fi}({\phi }_{F}^{in}-{\phi }_{F}^{out})=0$$

The DH chemical potential ${\mu }_{DH}$ is^62^

4 $${\mu }_{DH}({\phi }_{I}^{in,out})=-\frac{C{z}^{2}{e}^{2}}{2D}\left(\frac{\kappa }{1+\kappa b}\right)\simeq -\frac{C{z}^{2}{e}^{2}}{2D}\sqrt{\frac{2{F}^{2}}{D{\varepsilon }_{0}RT}I}=-A\sqrt{{\phi }_{I}^{in,out}}$$

In Eq.(4), I is the ionic strength of the salt solution defined as $\frac{1}{2}{\sum }_{i}{c}_{i}{z}_{i}^{2}$ where *c*_*i*_ and *z*_*i*_ are the concentration and valency of the *i*^*th*^ ionic species, D is the dielectric permittivity of water, F is Faraday’s constant ($=e{N}_{A}$), b is effective ion size, ${\varepsilon }_{0}$ is the vacuum permittivity, and $\kappa$ is the Debye length. At low to moderate salt concentrations $\kappa b\ll 1$ and we disregard this term for the qualitative analysis below.

Equivalently, the condition of equilibrium between FUS molecules in the bulk and condensate is that the chemical potential of FUS molecules in the condensate is equal to that in the bulk ${\mu }_{F}^{in}={\mu }_{F}^{out}$ which brings in the equation that determines the volume fraction of FUS in the condensate at a fixed volume fraction of FUS in the bulk:

5 $$\frac{1}{L}T\,{{{\mathrm{ln}}}}\left(\frac{{\phi }_{F}^{in}}{{\phi }_{F}^{out}}\right)-T\,{{{\mathrm{ln}}}}\left(\frac{1-{\phi }_{F}^{in}-{\phi }_{I}^{in}}{1-{\phi }_{F}^{out}-{\phi }_{I}^{out}}\right)+{\tilde{\chi }}_{FI}({\phi }_{I}^{in}-{\phi }_{I}^{out})+2{\tilde{\chi }}_{FF}({\phi }_{F}^{in}-{\phi }_{F}^{out})=0$$

Where volume fractions of ions in the bulk and the condensate are determined by the equilibrium condition Eq. (3).

Now we explore the whole range of densities for all components. To that end, in Fig. 5b we plot Eq. (3) for the difference of chemical potentials, $\Delta {\mu }_{I}$ *of ions* between condensed and dilute phases as a function of volume fraction of ions inside the condensate ${\phi }_{I}^{in}$ at various values of volume fractions of ions outside the condensate ${\phi }_{I}^{out}$. To illustrate the qualitative behavior of the chemical potential plots, we fix the volume fraction of FUS in the dilute phase, ${\phi }_{F}^{out}$ at 0.,1 and inside the condensate, ${\phi }_{F}^{in}$ at 0.3 (other values give qualitatively similar results).

We see from Fig. 5b that at a low salt concentration regime, the condensate strongly absorbs ions. The effect is largely due to the Debye-Hückel (DH) correlation, as the cancellation of the DH energy in the free energy functional shown in Eq.(2) greatly diminishes due to absorption of ions in the condensate (blue curve). However, as the bulk salt concentration increases, this effect diminishes and finally reverses, whereby the ions are depleted inside FUS condensate compared to the bulk. This can be summarized in Fig. 5c that shows the dependence of ion concentration inside FUS condensate on the ion concentration in the bulk at a fixed density of FUS condensate. We note the non-monotonic dependence of concentration of the ions with full charge interactions inside the condensate (red line). In contrast, in the absence of the charge-induced DH contribution to free energy, the non-monotonic dependence disappears. Rather, a much weaker monotonic dependency is observed (blue line in Fig. 5c). For further analysis of the ion equilibrium, including the limits on the reentrant behavior, see the **Supporting Information** text.

Finally, we solve Eqs. (3) and (5) at a fixed volume fraction of FUS in the dilute phase and obtain the equilibrium density (volume fraction) of FUS in the condensate in a broad range of bulk ion concentrations. The resulting typical plot (Fig. 5d) indeed shows reentrance and second transition at a higher concentration of ions, exactly as observed in experiments^26^. As an important control we explore a hypothetical situation where ion charges are abrogated [A = 0 in Eqs. (3)–(5)]. As seen on Fig. 5e the reentrant LLPS transition at low salt concentration disappears while the transition at higher salt remains unaffected, exactly as observed in simulations (Fig. 1).

Finally, to get a deeper insight into the origin of the low and high salt transitions we explore temperature dependence of both (Fig. 5f). As can be seen clearly, in a condensed phase formed at lower concentration of salt only the first reentrant transition occurs at low temperatures but the second transition at high salt appears at higher temperature and becomes stronger as temperature increases while the low salt transition becomes weaker and finally disappears as temperature increases. At intermediate temperatures, both transitions occur in their respective ranges of salt concentration. From that, we conclude that the first transition is driven by the energetics of FUS-ion and ion-ion interactions and is opposed by the unfavorable entropy of redistributing ions inside/outside the condensate, while the second transition is clearly driven by entropy that favors more uniform distributions of all components of the complex solution inside the condensate at high salt concentrations.

In summary, the predictions from analytical theory are:

1. The reentrant transition at low ionic strengths is driven by ionic charges, whereby FUS monomers effectively interact through the medium of highly correlated salt ions. In this regime ion concentration inside the condensate exceeds that of the bulk solution.
2. At a higher ionic strength, charge correlations become short-range, and the FUS condensate dissolves.
3. At even higher (non-physiological) ionic strengths, another FUS condensation transition, driven by entropy of mixing, occurs. In this regime, ionic charges do not play a crucial role, and the solvent components redistribute in the opposite direction, making the concentration of ions inside the condensate lower than in the bulk. This transition is akin to a collapse transition in a mixed solvent studied by Xia et al. ^58^.

## Discussion

Studies of charged polymers (polyelectrolytes and polyampholytes) in solutions containing salts have a long history^33–35,63–66^. Discovery of membrane-less organelles in living cells formed by IDPs undergoing LLPS^26^ reignited interest in biopolymer condensates in physiologically relevant environments^67^. Statistical mechanical analysis using field-theoretical tools from polymer theory^68,69^ provided detailed phase diagrams for specific IDPs enriched in charged amino acids^36^. In these cases, salts affect the phase state of a polyelectrolyte by screening electrostatic repulsion between its charges giving rise, in some cases, to LLPS. However, coacervation experiments did not reveal salt-dependent re-entrant transition^70^. In a similar vein, coacervation of polyelectrolytes of opposite charges appeared to be strongly salt-dependent as electrostatics is a driving force in these situations^65,70^.

Polyampholytes can form condensates without the presence of any ions (that is, at 0.0 M salt) as shown in Fig. S8, consistent with earlier studies^61^. In contrast, FUS-LCD-type uncharged sequences cannot form condensates without salt ions present. For polyampholytes, the presence of salt screens attractive interactions between charges, leading to de-condensation transition at higher salt concentration. In the case of the FUS-LCD type uncharged IDP, salt ions *drive* condensation. In the case of polyampholytes, the driver of condensation is the charges on the IDP. Turning off the charges of the salt ions does not dissolve the polyampholyte condensate, which is also different from the behavior shown by the FUS-LCD condensate. Apparently, the physics of condensation transition in globally neutral polyampholytes and salt-induced transition in uncharged IDPs are drastically different. Further discussion of this point is presented in the **Supporting Information** (Section 8). Recent studies on neutral, hydrophobic polymers have shown that polyanions like ATP can induce non-monotonic phase behavior through salting-out effects at low concentrations, followed by hydrotropic dissolution^71^. While this resembles the first re-entrant transition observed in our study, the mechanism we describe for polar uncharged IDPs like FUS-LCD is distinct. Unlike hydrophobic aggregation through ‘salting-out’, the phase separation at low ionic strength in FUS-LCD is driven by an ‘ion-correlated’ attraction where ions are partitioned into the condensate.

Recent experiments showed that largely uncharged IDPs such as FUS, TDP-43, and others undergo a set of complex reentrant transitions in a broad range of salt concentration^32^. The authors of ref. ^32^ first carried out a series of atomistic simulations to demonstrate that different forces are dominant at different salt concentrations. While insightful, these studies do not provide a clear mechanistic explanation of the multiple transitions that FUS and other uncharged IDPs undergo in a broad range of salt concentrations with multiple instances of reentrance. We further note that Krainer et al. employed the implicit solvent CG force field (no bead representation for ions and water) to simulate condensates, by tweaking the relative contribution of electrostatic and hydrophobic interactions among amino acid pairs, according to the salt concentration, based on their atomistic PMF results^32^. Therefore, in its original form with Debye-Hückel electrostatics and no explicit ions/water, the CG model they used would be incapable of capturing the reentrance. On the other hand, MARTINI can capture the reentrant without the need for salt-concentration specific parameterization, probably due to its explicit description of ions and water. The latter also reveals the spatial distribution of ions, which was not captured through implicit ion/solvent simulations.

In this work, we used a combination of theoretical and computational approaches to reach a mechanistic understanding of the complex reentrant phase behavior of uncharged IDPs at different salt concentrations. The mechanistic picture emerging from these analyses is as follows. At low salt concentrations, LJ interaction between an uncharged FUS monomer and a salt ion creates a local density perturbation, creating a density gradient in a highly correlated ion plasma medium that extends up to the Debye length. Given that interactions between ions and FUS monomers are energetically favorable due to LJ attraction, the excess of ion density created by one FUS monomer, which propagates up to the correlation length in the ion media, serves as an effective energetic funnel creating a long-range attractive potential for another FUS monomer(s). This effective long-range attraction between uncharged FUS monomers is illustrated in Fig. 2, which shows the PMF between two FUS monomers in the ionic media derived from the MARTINI simulation. In the full model, where ion charges are intact, the PMF shows effective long-range attractive interactions that extend up to ~10 nm length scale at physiological salt concentration (black line in Fig. 2). When ion charges are abrogated or only one kind of ion is present, this effective attractive potential disappears (red and green lines in Fig. 2).

This mechanism is conceptually similar to polymer-salt-induced condensation, often referred to as ψ-condensation^59^. *ψ*-condensation refers to the collapse of DNA in the presence of an uncharged polymer (polyethylene oxide, PEO) and monovalent salt in solution^72^. In $\psi$-condensation, DNA-induced perturbation of the density of PEO propagates at longer scales due to correlated fluctuations in polymer solution, creating an effective long-range potential well for DNA^59^ – a mechanism similar to the one described here, where the role of correlated medium is played by ions.

As salt concentration increases beyond physiological levels, screening effects become increasingly significant, leading to a suppression of long-range attractive interactions and, ultimately, dissolution of the condensate. Our results show that this dissolution occurs around between 1 M – 2 M salt concentration. However, an intriguing phenomenon emerges at even higher ionic strengths, where LLPS reappears beyond 2 M salt concentration, marking the second reentrant transition. Unlike the condensation mechanism at low salt, phase separation in this regime is not driven by FUS-salt interactions but rather by an ion-exclusion mechanism akin to depletion forces. The FUS condensate expels ions from its interior, allowing the system to regain phase separation despite the high salt environment. The second LLPS transition at high salt concentration is an entropy driven phenomenon which disappears at low temperature as predicted by the theory (see Fig. 5e). CG simulations show the same behavior: while transition at low salt is crucially dependent on ion charges the second transition at high salt is insensitive to the abrogation of charges of salt ions suggesting that it is driven mainly by LJ energy of association between FUS and salt and, crucially entropy of all components in the condensate^58^.

This is a striking departure from the behavior observed at low ionic strength, underscoring the nontrivial role of electrostatic correlations and entropic forces in governing phase behavior in IDPs with a low fraction of charged residues. A key insight from our study is that the mechanism of LLPS at low and high salt concentrations is fundamentally distinct, even though both regimes exhibit phase separation. This distinction is evident in the differences in ion distribution, pair interaction energies, and overall condensate morphology. The ψ-condensation-like mechanism at physiological salt concentrations leads to a system where ions are deeply embedded in the condensate, while at extreme salt conditions, the driving force shifts towards an entropic phase separation driven by ion exclusion. These findings not only provide a microscopic understanding of the reentrant LLPS of FUS but also suggest a generalizable framework for other IDPs with low charge density. However, whether structured domains follow the same physics or require a modified treatment remains to be understood.

Overall, our study provides a comprehensive statistical mechanical view of mechanisms of LLPS in water-salt solutions, where theoretical predictions are tested and verified by molecular simulations. By unraveling the mechanistic details of LLPS across different ionic environments, we not only explain recent experimental observations but also establish a broader paradigm for understanding phase separation in biologically relevant uncharged systems.

## Supplementary information

Transparent Peer Review file

Supporting information

## Data Availability

All relevant data and simulation files (including trajectories) can be accessed from 10.5281/zenodo.18727726. Any other information is available from the authors upon reasonable requests.

## References

[1] Hyman, A. A., Weber, C. A. & Jülicher, F. Liquid-liquid phase separation in biology. *Annu. Rev. Cell Dev. Biol.* **30**, 39–58 (2014). 25288112 10.1146/annurev-cellbio-100913-013325

[2] Banani, S. F., Lee, H. O., Hyman, A. A. & Rosen, M. K. Biomolecular condensates: organizers of cellular biochemistry. *Nat. Rev. Mol. Cell Biol.* **18**, 285–298 (2017). 28225081 10.1038/nrm.2017.7 PMC7434221

[3] Ladouceur, A.-M. et al. Clusters of bacterial RNA polymerase are biomolecular condensates that assemble through liquid–liquid phase separation. *Proc. Natl. Acad. Sci.* **117**, 18540–18549 (2020). 32675239 10.1073/pnas.2005019117 PMC7414142

[4] Laflamme, G. & Mekhail, K. Biomolecular condensates as arbiters of biochemical reactions inside the nucleus. *Commun. Biol.* **3**, 773–773 (2020). 33319830 10.1038/s42003-020-01517-9 PMC7738674

[5] Gibson, B. A. et al. Organization of chromatin by intrinsic and regulated phase separation. *Cell* **179**, 470–484 (2019). 31543265 10.1016/j.cell.2019.08.037 PMC6778041

[6] Guillén-Boixet, J. et al. RNA-induced conformational switching and clustering of G3BP drive stress granule assembly by condensation. *Cell* **181**, 346–361 (2020). 32302572 10.1016/j.cell.2020.03.049 PMC7181197

[7] Klosin, A. et al. Phase separation provides a mechanism to reduce noise in cells. *Science* **367**, 464–468 (2020). 31974256 10.1126/science.aav6691

[8] Case, L. B., Ditlev, J. A. & Rosen, M. K. Regulation of transmembrane signaling by phase separation. *Annu. Rev. Biophys.* **48**, 465–494 (2019). 30951647 10.1146/annurev-biophys-052118-115534 PMC6771929

[9] Mondal, S. & Cui, Q. Coacervation-Induced Remodeling of Nanovesicles. *J. Phys. Chem. Lett*. 10.1021/acs.jpclett.3c00705 (2023) 10.1021/acs.jpclett.3c00705 37159305

[10] Mangiarotti, A., Chen, N., Zhao, Z., Lipowsky, R. & Dimova, R. Wetting and complex remodeling of membranes by biomolecular condensates. *Nat. Commun.* **14**, 2809–2809 (2023). 37217523 10.1038/s41467-023-37955-2 PMC10203268

[11] Alberti, S. & Dormann, D. Liquid–liquid phase separation in disease. *Annu. Rev. Genet.* **53**, 171–194 (2019). 31430179 10.1146/annurev-genet-112618-043527

[12] Pak, C. W. et al. Sequence determinants of intracellular phase separation by complex coacervation of a disordered protein. *Mol. Cell* **63**, 72–85 (2016). 27392146 10.1016/j.molcel.2016.05.042 PMC4973464

[13] Abyzov, A., Blackledge, M. & Zweckstetter, M. Conformational dynamics of intrinsically disordered proteins regulate biomolecular condensate chemistry. *Chem. Rev.* **122**, 6719–6748 (2022). 35179885 10.1021/acs.chemrev.1c00774 PMC8949871

[14] Dinic, J., Marciel, A. B. & Tirrell, M. V. Polyampholyte physics: Liquid–liquid phase separation and biological condensates. *Curr. Opin. Colloid Interface Sci.* **54**, 101457–101457 (2021).

[15] Kaur, T. et al. Sequence-encoded and composition-dependent protein-RNA interactions control multiphasic condensate morphologies. *Nat. Commun.* **12**, 872–872 (2021). 33558506 10.1038/s41467-021-21089-4 PMC7870978

[16] McCarty, J., Delaney, K. T., Danielsen, S. P. O., Fredrickson, G. H. & Shea, J.-E. Complete phase diagram for liquid–liquid phase separation of intrinsically disordered proteins. * J. Phys. Chem. Lett.* **10**, 1644–1652 (2019). 30873835 10.1021/acs.jpclett.9b00099 PMC7379843

[17] Ranganathan, S. & Shakhnovich, E. I. Dynamic metastable long-living droplets formed by sticker-spacer proteins. *Elife* **9**, e56159–e56159 (2020). 32484438 10.7554/eLife.56159 PMC7360371

[18] Ranganathan, S., Dasmeh, P., Furniss, S. & Shakhnovich, E. Phosphorylation sites are evolutionary checkpoints against liquid–solid transition in protein condensates. *Proc. Natl. Acad. Sci.* **120**, e2215828120–e2215828120 (2023). 37155880 10.1073/pnas.2215828120 PMC10193986

[19] Chattaraj, A. & Shakhnovich, E. I. Separation of sticker-spacer energetics governs the coalescence of metastable condensates. *Biophys. J.* **124**, 28–439 (2025). 10.1016/j.bpj.2024.12.017 PMC11788481 39674888

[20] Wang, J. et al. A molecular grammar governing the driving forces for phase separation of prion-like RNA binding proteins. *Cell* **174**, 688–699 (2018). 29961577 10.1016/j.cell.2018.06.006 PMC6063760

[21] Saar, K. L. et al. Learning the molecular grammar of protein condensates from sequence determinants and embeddings. *Proc. Natl. Acad. Sci.* **118**, e2019053118–e2019053118 (2021). 33827920 10.1073/pnas.2019053118 PMC8053968

[22] Brangwynne, C. P., Tompa, P. & Pappu, R. V. Polymer physics of intracellular phase transitions. *Nat. Phys.* **11**, 899–904 (2015).

[23] Li, P. et al. Phase transitions in the assembly of multivalent signalling proteins. *Nature* **483**, 336–340 (2012). 22398450 10.1038/nature10879 PMC3343696

[24] Ahlers, J. et al. The key role of solvent in condensation: Mapping water in liquid-liquid phase-separated FUS. *Biophys. J.* **120**, 1266–1275 (2021). 33515602 10.1016/j.bpj.2021.01.019 PMC8059208

[25] Mukherjee, S. & Schäfer, L. V. Thermodynamic forces from protein and water govern condensate formation of an intrinsically disordered protein domain. *Nat. Commun.* **14**, 5892–5892 (2023). 37735186 10.1038/s41467-023-41586-y PMC10514047

[26] Berry, J., Brangwynne, C. P. & Haataja, M. Physical principles of intracellular organization via active and passive phase transitions. *Rep. Prog. Phys.* **81**, 046601–046601 (2018). 29313527 10.1088/1361-6633/aaa61e

[27] Lee, D. S. W. et al. Size distributions of intracellular condensates reflect competition between coalescence and nucleation. *Nat. Phys.* **19**, 586–596 (2023). 37073403 10.1038/s41567-022-01917-0 PMC10104779

[28] Das, R. K. & Pappu, R. V. Conformations of intrinsically disordered proteins are influenced by linear sequence distributions of oppositely charged residues. *Proc. Natl. Acad. Sci.* **110**, 13392–13397 (2013). 23901099 10.1073/pnas.1304749110 PMC3746876

[29] Monahan, Z. et al. Phosphorylation of the FUS low-complexity domain disrupts phase separation, aggregation, and toxicity. * EMBO J.* **36**, 2951–2967 (2017). 28790177 10.15252/embj.201696394 PMC5641905

[30] Narayanan, T. & Kumar, A. Reentrant phase transitions in multicomponent liquid mixtures. *Phys. Rep.* **249**, 135–218 (1994).

[31] Dignon, G. L., Zheng, W., Kim, Y. C., Best, R. B. & Mittal, J. Sequence determinants of protein phase behavior from a coarse-grained model. *PLoS Comput. Biol.* **14**, e1005941–e1005941 (2018). 29364893 10.1371/journal.pcbi.1005941 PMC5798848

[32] Krainer, G. et al. Reentrant liquid condensate phase of proteins is stabilized by hydrophobic and non-ionic interactions. *Nat. Commun.* **12**: 1085. *Nat. Commun.* **12** (2021). 10.1038/s41467-021-21181-9 PMC7889641 33597515

[33] Srivastava, S. & Tirrell, M. V. Polyelectrolyte complexation. *Adv. Chem. Phys.* **161**, 499–544 (2016).

[34] Michaeli, I., Overbeek, J. T. G. & Voorn, M. J. Phase separation of polyelectrolyte solutions. *J. Polym. Sci.* **23**, 443–450 (1957).

[35] Sing, C. E. & Perry, S. L. Recent progress in the science of complex coacervation. *Soft Matter* **16**, 2885–2914 (2020). 32134099 10.1039/d0sm00001a

[36] Lin, Y.-H. et al. Electrostatics of salt-dependent reentrant phase behaviors highlights diverse roles of ATP in biomolecular condensates. *eLife* **13**, RP100284–RP100284 (2025). 40028898 10.7554/eLife.100284 PMC11875540

[37] Zhang, P., Shen, K., Alsaifi, N. M. & Wang, Z.-G. Salt partitioning in complex coacervation of symmetric polyelectrolytes. *Macromolecules* **51**, 5586–5593 (2018).

[38] Souza, P. C. T. et al. Martini 3: a general purpose force field for coarse-grained molecular dynamics. *Nat. methods* **18**, 382–388 (2021). 33782607 10.1038/s41592-021-01098-3 PMC12554258

[39] Tsanai, M., Frederix, P. W. J. M., Schroer, C. F. E., Souza, P. C. T. & Marrink, S. J. Coacervate formation studied by explicit solvent coarse-grain molecular dynamics with the Martini model. *Chem. Sci.* **12**, 8521–8530 (2021). 34221333 10.1039/d1sc00374g PMC8221187

[40] Mehta, N., Mondal, S., Watson, E. T., Cui, Q. & Chapman, E. R. The juxtamembrane linker of synaptotagmin 1 regulates Ca2+ binding via liquid-liquid phase separation. *Nat. Commun.* **15**, 262–262 (2024). 38177243 10.1038/s41467-023-44414-5 PMC10766989

[41] Mondal, S. & Cui, Q. Sequence Sensitivity in Membrane Remodeling by Polyampholyte Condensates. * J. Phys. Chem. B* **128**, 2087–2099 (2024). 38407041 10.1021/acs.jpcb.3c08149

[42] Thomasen, F. E., Pesce, F., Roesgaard, M. A., Tesei, G. & Lindorff-Larsen, K. Improving Martini 3 for disordered and multidomain proteins. *J. Chem. Theory Comput.* **18**, 2033–2041 (2022). 35377637 10.1021/acs.jctc.1c01042

[43] Benayad, Z., von Bülow, S., Stelzl, L. S. & Hummer, G. Simulation of FUS protein condensates with an adapted coarse-grained model. *J. Chem. Theory Comput.* **17**, 525–537 (2020). 33307683 10.1021/acs.jctc.0c01064 PMC7872324

[44] Zerze, G. H. Optimizing the martini 3 force field reveals the effects of the intricate balance between protein–water interaction strength and salt concentration on biomolecular condensate formation. *J. Chem. Theory Comput.* **20**, 1646–1655 (2023). 37043540 10.1021/acs.jctc.2c01273

[45] Zinga, K. et al. Simulations reveal a balance between protein–protein and protein–lipid interactions during condensation on membrane surfaces. *Chem. Sci.* **16**, 18869–18883 (2025). 10.1039/d5sc04862a PMC12439209 40963550

[46] UniProt: the universal protein knowledgebase in 2021. *Nucleic Acids Res.* **49**, D480–D489 (2021). 10.1093/nar/gkaa1100 PMC7778908 33237286

[47] DeLano, W. L. Pymol: An open-source molecular graphics tool. *CCP4 Newsl. Protein Crystallogr.* **40**, 82–92 (2002).

[48] Kroon, P. C. et al. Martinize2 and vermouth: Unified framework for topology generation. *eLife* **12**, RP90627 (2023). 10.7554/eLife.90627 PMC12634043 41263305

[49] Bussi, G., Donadio, D. & Parrinello, M. Canonical sampling through velocity rescaling. *J. Chem. Phys.* **126**, 014101 (2007). 10.1063/1.2408420 17212484

[50] Bernetti, M. & Bussi, G. Pressure control using stochastic cell rescaling. *J. Chem. Phys.* **153**, 114107 (2020). 10.1063/5.0020514 32962386

[51] Abraham, M. J. et al. GROMACS: High performance molecular simulations through multi-level parallelism from laptops to supercomputers. *SoftwareX* **1**, 19–25 (2015).

[52] Tribello, G. A., Bonomi, M., Branduardi, D., Camilloni, C. & Bussi, G. PLUMED 2: New feathers for an old bird. *Comput. Phys. Commun.* **185**, 604–613 (2014).

[53] Humphrey, W., Dalke, A. & Schulten, K. VMD: visual molecular dynamics. *J. Mol. Graph.* **14**, 33–38 (1996). 8744570 10.1016/0263-7855(96)00018-5

[54] Barducci, A., Bussi, G. & Parrinello, M. Well-tempered metadynamics: a smoothly converging and tunable free-energy method. *Phys. Rev. Lett.* **100**, 020603 (2008). 18232845 10.1103/PhysRevLett.100.020603

[55] Raos, G. & Allegra, G. Chain collapse and phase separation in poor-solvent polymer solutions: A unified molecular description. * J. Chem. Phys.* **104**, 1626–1645 (1996).

[56] Farag, M. et al. Condensates formed by prion-like low-complexity domains have small-world network structures and interfaces defined by expanded conformations. *Nat. Commun.* **13**, 7722 (2022). 36513655 10.1038/s41467-022-35370-7 PMC9748015

[57] Wang, J., Devarajan, D. S., Nikoubashman, A. & Mittal, J. Conformational properties of polymers at droplet interfaces as model systems for disordered proteins. *ACS Macro Lett.* **12**, 1472–1478 (2023). 37856873 10.1021/acsmacrolett.3c00456 PMC10771815

[58] Xia, Z., Das, P., Shakhnovich, E. I. & Zhou, R. Collapse of unfolded proteins in a mixture of denaturants. *J. Am. Chem. Soc.* **134**, 18266–18274 (2012). 23057830 10.1021/ja3031505

[59] Grosberg, A. Y., Erukhimovitch, I. Y. & Shakhnovich, E. I. On the Theory of Ψ-Condensation. *Biopolymers* **21**, 2413–2432 (1982).

[60] Ingólfsson, H. I. et al. Multiscale simulations reveal TDP-43 molecular-level interactions driving condensation. *Biophys. J.* **122**, 4370–4381 (2023). 37853696 10.1016/j.bpj.2023.10.016 PMC10720261

[61] Wessén, J., Pal, T. & Chan, H. S. Field theory description of ion association in re-entrant phase separation of polyampholytes. *The J. Chem. Phys.* **156**, 194903 (2022). 10.1063/5.0088326 35597632

[62] McQuarrie, D. A. *Statistical Mechanics*. (University Science Books, 2000).

[63] Kudlay, A. & Olvera de la Cruz, M. Precipitation of oppositely charged polyelectrolytes in salt solutions. * J. Chem. Phys.* **120**, 404–412 (2004). 15267302 10.1063/1.1629271

[64] Danielsen, S. P. O., McCarty, J., Shea, J. E., Delaney, K. T. & Fredrickson, G. H. Small ion effects on self-coacervation phenomena in block polyampholytes. *J. Chem. Phys.* **151**, 034904 (2019). 31325933 10.1063/1.5109045 PMC6639116

[65] Borue, V. Y. & Erukhimovich, I. Y. A statistical theory of weakly charged polyelectrolytes: fluctuations, equation of state and microphase separation. *Macromolecules* **21**, 3240–3249 (1988).

[66] Borue, V. Y. & Erukhimovich, I. Y. A statistical theory of globular polyelectrolyte complexes. *Macromolecules* **23**, 3625–3632 (1990).

[67] Baidya, L., Maity, H. & Reddy, G. Salts Influence IDP properties by modulating the population of conformational clusters. *J. Phys. Chem. B.* **129**, 2433–2445 (2025). 10.1021/acs.jpcb.4c08248 39977663

[68] Lin, Y.-H., Brady, J. P., Chan, H. S. & Ghosh, K. A unified analytical theory of heteropolymers for sequence-specific phase behaviors of polyelectrolytes and polyampholytes. *J. Chem. Phys.* **152**, 045102 (2020). 10.1063/1.5139661 PMC7043852 32007034

[69] Najafi, S., McCarty, J., Delaney, K. T., Fredrickson, G. H. & Shea, J. E. Field-theoretic simulation method to study the liquid-liquid phase separation of polymers. *Methods Mol. Biol.* **2563**, 37–49 (2023). 36227467 10.1007/978-1-0716-2663-4_2

[70] Priftis, D. & Tirrell, M. Phase behaviour and complex coacervation of aqueous polypeptide solutions. *Soft Matter* **8**, 9396–9405 (2012).

[71] Ayvaz, C., Ozdogan, Y. S., Peker, D. S., Erbaş, A. & Okur, H. I. ATP can act as a stabilizer on neutral macromolecules. * J. Phys. Chem. Lett.* **16**, 10771–10777 (2025). 41056468 10.1021/acs.jpclett.5c02467 PMC12536438

[72] Lerman, L. S. A transition to a compact form of DNA in polymer solutions. *Proc. Natl. Acad. Sci.* **68**, 1886–1890 (1971). 5288774 10.1073/pnas.68.8.1886 PMC389314

