# Supplementary material for: Origin of the ionic-strength dependent reentrant behavior in the liquid-liquid phase separation of uncharged intrinsically disordered proteins
Source: Commun Chem. 2026 Apr 11;9:210. doi: 10.1038/s42004-026-02011-9 (PMC13269793; doi:10.1038/s42004-026-02011-9)
Supplement: Supplementary file 1 — Transparent Peer Review file [file 42004_2026_2011_MOESM1_ESM.pdf]

nature portfolio

Peer Review File

Origin of the Ionic-strength Dependent Reentrant Behavior in the Liquid-Liquid Phase Separation of Uncharged Intrinsically Disordered Proteins

Corresponding Author: Professor Eugene Shakhnovich

This file contains all reviewer reports in order by version, followed by all author rebuttals in order by version.

Version 0:

Reviewer comments:

Reviewer #1

(Remarks to the Author)
The authors investigated the molecular mechanisms of the reentrant phase behavior of uncharged IDPs by combining coarse-grained simulations and analytical theories. The results provide interesting and new insights into the mechanisms which go beyond the traditional understanding of the influence of ions on phase separation or coacervation. While the manuscript makes a valuable contribution to the field, it can be published after addressing the following concerns.

1. The authors mention that “However, this explanation is not applicable to mostly uncharged IDPs such as FUS and TDP-43 …” in page 4, and Table S1a lists several sequences for which the authors claim that the reentrant phase behavior cannot be explained by the mechanisms reported in previous studies. However, the fraction of charged residues for all sequences in Table S1a is around 20%, which is comparable to that of LAF-1 protein that undergoes phase separation driven by electrostatic interactions. This suggests that the electrostatic interactions, at least in part, could contribute significantly to the phase separation of these proteins. Consequently, the conventional explanation of the effect of salt on phase separation might still be applicable to these proteins. I understand that the statement servers as the motivation for the study and do not affect the subsequent results, but these statements seems somewhat overstated. The authors may consider rephrasing these statements for clarity and accuracy.

2. For the contact order parameter defined in Eq. 1, is there any specific reason or physical rationale for using this equation instead of contacting numbers defined by a cutoff distance? A brief explanation of the choice would improve clarity.

3. Since the interactions between proteins and ions are crucial, the relevant force field parameters, especially those for the salts are suggested to listed in SI.

4. Polymer theories and molecular simulations have shown that single chains within the condensates should be expanded, the Rg value is higher than those in the corresponding dilute phase, and the scaling exponent is near 0.6, similar to those for self-avoiding walks, see 1) Fig. 10 in Raos and Allegra, J. Chem. Phys. 104, 1626–1645 (1996); 2) Fig. 2 in M. Farag, Nat. Commu., 2022, 13:7722; 3) Fig. 5 in ACS Macro Lett. 2023, 12, 1472–1478. However, Fig. 1b shows that non-phase-separating systems have much higher Rg values than those in phase separating systems, which is unexpected. I suspect that the finite size effect may significantly influence the reported Rg values in Fig 1c as there’re only 25 copies of full-length FUS in the simulation box. Considering that the full-length FUS is quite long with 500+ residues, it is possible that the Rg value of the expanded conformation of a single-chain FUS is larger than the radius of the small “droplet” formed by the 25 copies. The authors should investigate this potential issue and include a short discussion if the results of Rg values are retained in the revised manuscript.

5. Followed by the above question, the authors should discuss the potential impact of the finite-size effects in this study or demonstrate that these effects do not affect the major conclusions.

6. Error bar should be added to the PMF curves in Fig. 2 and supporting data should be provided to show the convergence of the sampling.

7. Fig 3b shows that the artificial ‘chargeless’ FUS chains have phase separation at low salt concentration, and the authors attribute this to the interactions through highly correlated ionic plasma in Page 13. It seems that this is quite a general phenomenon, namely non-charged disordered proteins can undergo phase separation at low salt concentration. Can the authors have some discussions on the generalizability of this phenomenon? For example, does this phenomenon depend on the specific sequences? Additionally, how might the coarse-grained parameters for salts influence these observations?

8. The authors mentioned that “the PMF shows effective long-range attractive interactions that extend up to 12 nm length scale” in Page 26, this may be only due to the fact that the FUS protein is quite long and there are still some direct contacts when their COM distance is beyond than 12nm.

9. Since analytical theory is used, is that possible to compare the enthalpy energies in the system before and after phase separation, as well as the entropy part? This will provide direct evidence for the driving force to the phase separation and support the major conclusions.

Reviewer #2

(Remarks to the Author)
The manuscript combines computational (coarse-grained models in explicit solvent) and analytical methods at the mean-field level to describe the salt-dependent assembly and disassembly of FU, an intrinsically disordered protein. The authors argue that at physiologically relevant salt concentrations, ion correlations can form long-range attractions (~10 nm), which in turn lead to the aggregation of FUS. At higher salt levels, the aggregate disassembles, and eventually, at excess salt concentration (>1 M), an aggregate formation (similar to that observed at lower salt levels) reappears. The manuscript focuses on a very important aspect of coacervate formation and convincingly combines analytical and computational tools to explain how electrostatics can control the phase segregation of charge-neutral molecules. In my opinion, this timely manuscript is suitable for publication after some minor revisions.

Major comments:
- Line 99: The Voorn -Overbreek transition can be explained in the main text briefly
- I would assume that no long-range electrostatics were calculated in the simulations, which is reasonable given the high salt concentration. However, the manuscript can benefit from benchmarking regarding the cut-off distance (1.1 nm).
- What is the underlying reason for choosing the dielectric constant 15?
- What do the Authors think of the effect of the water dipole, which is, I believe, absent in Martini water models?
- Line 416, the first sentence of the paragraph, appears to be inconsistent.
- The ATP, which has a net charge and its own counterion (effectively a salt), has been identified as a hydrotropic molecule that affects the solvation properties of protein coacervates (Patel et al., Science, 2017). A recent study has shown that ATP can also achieve the same function, but on neutral polymers (Ayvaz et al., J. Phys. Chem. Lett., 2025). These experiments and simulations reported a non-monotonic behavior on the conformation of neutral chains as a function of ATP. Can the Authors comment on the possible effect of their results on the hydrotropic properties of ATP?

Minor comments:
- Snapshots in Figure 1 can be larger.
- Line 230, the Authors still call the charge-neutral NA and Cl beads “salt”.
- Line 265/266, typos.
- Does Eq.2 miss the Boltzmann constant, given that T is the absolute temperature defined in the Methods section?
- Figure 5b shows the salt concentration in the figure titles
- Figures 5d-f have a lower quality than other figures, and Figure 5f misses the right-aligned axis.

Reviewer #3

(Remarks to the Author)
The work of Mondal & Shakhnovich is probably the most interesting paper on biomolecular condensates that I have read in a while and will be an essential contribution in the field, providing a clear and generalizable explanation for the salt dependent phase separation of uncharged or almost uncharged disordered proteins. The manuscript is thought provoking and provides not only a computational model but a theoretical framework that enables dissecting the various aspect involved in the studied re-entrant phase behavior. The results are particularly important because they also demonstrate the importance of properly accounting for ions in simulations, which is normally neglected in many of the typical coarse-grained simulations, potentially affecting interpretation of driving forces and even properties of the dense phase.
I don’t have concerns but only few suggestions and curiosity that I help may improve the manuscript and perhaps the accessibility of results – I think that while the results are grounded in polymer physics, they are of essential importance for any scientist wanting to understand the interactions at play in biomolecular condensates.
I leave to the authors addressing any of my suggestion or curiosity, if they think they could improve the manuscript.
Suggestions/curiosity:
1. I think it would be helpful to define, if possible, a threshold in the DQ/N parameter to qualify phase separation vs small cluster formation. It would facilitate the interpretation of Figure 1 re-entrant phase diagram. I do agree the radius of gyration provides a strong qualifier, but it would add some sort of empirical boundaries that are helpful in the visualization of which phase occurs at which concentration.

2. Line 227-228: “we note that these Rg… taken together … and do not refer to single chain Rg”. Can the author clarify this sentence? How are the Rg computed and what they do represent?
3. Looking at the long-range effect of the potential of mean force between two FUS protein in Figure 2, I wonder whether the conformations of FUS molecules (e.g. the IDR region) are uniform or vary with salt concentration. It would be informative for future experimental attempts to provide simulated observables for single chains end-to-end and internal distances across the condensate to address if the effect of interaction is uniform across all FUS IDRs and whether it does impact equally the overall sequence or specific internal distances.
4. Line 265-266: two typos. Predcited instead of predicted
5. Line 373. Eq: 4, typo (a square appears instead of a mathematical symbol
6. Figure 5: panel b would benefit of a graphical highlighting that vertical dashed lines represent phi_I^OUT
7. I would suggest promoting Fig S9 in the main text because of the importance in the comparison of systems behavior. I wonder if the authors could consider adding also some comments on phase separation of polyelectrolyte systems in high salt, as shown for nucleic acids – if it would operate according a similar depletion mechanism.
8. This brings me to my personal curiosities that may be of interest to others to. Is the depletion effect at high molar salt true for all sequences? And is the disordered nature of the sequence relevant to it or would occur also for simply rigid domains? I think it would be important to explicit how generalizable is the system and what is the importance of being disordered or not.
9. Can one think about polyampholyte charges (and perhaps polyelectrolyte) as disruptors of the ion correlations in the ion plasma?
10. In general, would additional components partitioning with FUS in the cell disrupt the ion plasma? I understand this goes beyond this work and may be an avenue for future works, but some speculation on the physiological context would be interesting.
11. What is the length scale of correlations that one can expect due to the ion plasma? And could they impact not only partitioning, but also organization or material properties of the condensate?

Version 1:

Reviewer comments:

Reviewer #1

(Remarks to the Author)
The authors have addressed all my concerns. Regarding their response to Question 9 of Reviewer 1, I do believe that it is possible to get the entropy/enthalpy for each component from the theory, at least numerical results can be obtained. In my opinion, that would provide additional physical insights into the phenomenon observed in this study. However, the paper remains solid even without entropy/enthalpy decomposition, and I believe it can be published now.

Reviewer #2

(Remarks to the Author)
The authors have improved their manuscript based my and other two referees' comments, and now the manuscript is suitable for publication.

Reviewer #3

(Remarks to the Author)
I thank the authors for addressing, where possible, my concerns. I have no further comments.

Open Access This Peer Review File is licensed under a Creative Commons Attribution 4.0 International License, which permits use, sharing, adaptation, distribution and reproduction in any medium or format, as long as you give appropriate credit to the original author(s) and the source, provide a link to the Creative Commons license, and indicate if changes were made.
In cases where reviewers are anonymous, credit should be given to 'Anonymous Referee' and the source.
The images or other third party material in this Peer Review File are included in the article's Creative Commons license, unless indicated otherwise in a credit line to the material. If material is not included in the article's Creative Commons license and your intended use is not permitted by statutory regulation or exceeds the permitted use, you will need to obtain permission directly from the copyright holder.
To view a copy of this license, visit https://creativecommons.org/licenses/by/4.0/

Reviewer #1

The authors investigated the molecular mechanisms of the reentrant phase behavior of uncharged IDPs by combining coarse-grained simulations and analytical theories. The results provide interesting and new insights into the mechanisms which go beyond the traditional understanding of the influence of ions on phase separation or coacervation. While the manuscript makes a valuable contribution to the field, it can be published after addressing the following concerns.

Thank you for the positive comments and for recognizing our efforts towards a better understanding of salt driven LLPS of uncharged IDPs. We have addressed the concerns below and modified the manuscript accordingly.

1. The authors mention that "However, this explanation is not applicable to mostly uncharged IDPs such as FUS and TDP-43 …" in page 4, and Table S1a lists several sequences for which the authors claim that the reentrant phase behavior cannot be explained by the mechanisms reported in previous studies. However, the fraction of charged residues for all sequences in Table S1a is around 20%, which is comparable to that of LAF-1 protein that undergoes phase separation driven by electrostatic interactions. This suggests that the electrostatic interactions, at least in part, could contribute significantly to the phase separation of these proteins. Consequently, the conventional explanation of the effect of salt on phase separation might still be applicable to these proteins. I understand that the statement serves as the motivation for the study and do not affect the subsequent results, but these statements seem somewhat overstated. The authors may consider rephrasing these statements for clarity and accuracy.

We thank the reviewer for this insightful observation regarding the charge fractions of FUS and TDP-43. Our goal was to distinguish the mechanistic drivers of reentrant LLPS in highly charged polyampholytes from those in sequences where the charge density is lower, and the sequence is dominated by polar or aromatic residues.

We comprehend that a ~20% charge fraction is comparable to proteins like LAF-1; however, our focus is specifically on the *low-complexity domains* (LCDs) of these proteins (as detailed in **Table S1b**), which exhibit distinct sequence features compared to classic polyampholytes. To avoid any overstatement and to improve clarity, we have rewritten the sentence in the revised manuscript (**page 4, lines 85-87**) as follows:

"However, the salt-dependent reentrant LLPS of uncharged IDPs such as the low-complexity domains of FUS and TDP-43 may not be fully captured by the conventional electrostatic models alone."

2. For the contact order parameter defined in Eq. 1, is there any specific reason or physical rationale for using this equation instead of contacting numbers defined by a cutoff distance? A brief explanation of the choice would improve clarity.

We thank the reviewer for this question regarding the choice of the contact order parameter. We chose the formulation in **Eq. 1** because it employs a continuous weighting function, often referred to as a switching function, rather than a discrete cutoff like the Heaviside step function. It can take any value in between depending on the pair distance as shown in the figure below:

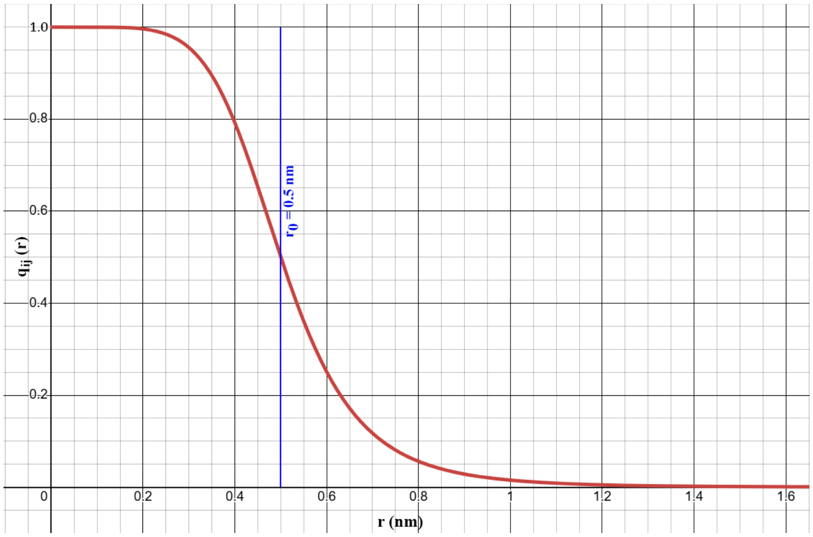

Figure R1. The variation of the switching function, q_{ij}(r) with distance (r).

While a binary cutoff assigns a value of 1 to any pair within a specified distance and 0 otherwise, our chosen formalism incorporates distance-dependent weights to provide a more nuanced representation of the structural ensemble.

This choice is grounded in several physical and computational rationales. First, continuous functions offer significant robustness to noise by being less sensitive to small thermal fluctuations near the threshold distance. In a binary model, a residue pair oscillating slightly around the cutoff- for example, between 4.9 Å and 5.1 Å- would cause the contact count to jump abruptly between 1 and 0, whereas our approach smooths these transitions and provides a more stable average. Second, this method aligns with physical realism, as intermolecular interactions do not "turn off" instantaneously at a specific distance but rather decay continuously; a weighted function better reflects this varying strength of interaction. Finally, continuous functions are mathematically differentiable, which is a distinct advantage for any subsequent analysis or potential optimization tasks involving the contact map.

We included the following sentences to the methods section (**page 9, lines 185-189**) for clarity. Thanks for the suggestion.

"Unlike discrete cutoff methods that utilize a Heaviside step function, Eq. (1) employs a continuous switching function to calculate contact orders. This approach provides a differentiable parameter that is more robust to small conformational fluctuations and better captures the distance-dependent nature of residue interactions."

3. Since the interactions between proteins and ions are crucial, the relevant force field parameters, especially those for the salts are suggested to listed in SI.

Thanks for the comment. The protein (FUS-LCD) is described by 15 unique bead types in the MARTINI3 force field. In addition, we have water (W) and ion (TQ5) bead types. Hence, there are ${}^{17}_{2}C = 136$ unique interaction pairs. We have now included the force-field file (name: **martini_3_lambda_1.03_fuslc.itp**) as a supplemental material that contains all the necessary information.

4. Polymer theories and molecular simulations have shown that single chains within the condensates should be expanded, the Rg value is higher than those in the corresponding dilute phase, and the scaling exponent is near 0.6, similar to those for self-avoiding walks, see 1) Fig. 10 in Raos and Allegra, J. Chem. Phys. 104, 1626–1645 (1996); 2) Fig. 2 in M. Farag, Nat. Commu., 2022, 13:7722; 3) Fig. 5 in ACS Macro Lett. 2023, 12, 1472−1478. However, Fig. 1b shows that non-phase-separating systems have much higher Rg values than those in phase separating systems, which is unexpected. I suspect that the finite size effect may significantly influence the reported Rg values in Fig 1c as there're only 25 copies of full-length FUS in the simulation box. Considering that the full-length FUS is quite long with 500+ residues, it is possible that the Rg value of the expanded conformation of a single-chain FUS is larger than the radius of the small "droplet" formed by the 25 copies. The authors should investigate this potential issue and include a short discussion if the results of Rg values are retained in the revised manuscript.

We thank the reviewer for this insightful comment regarding the scaling behavior of single chains within condensates. We completely agree that, based on polymer theory and recent literature, a single chain within a dense phase should exhibit an expanded conformation compared to its state in a dilute phase. However, we would like to clarify a possible misunderstanding regarding the data presented in **Figures 1b** and **1d**. **The Rg values plotted in these panels represent the global radius of gyration of all 42 FUS-LCD chains (163 residues each) within the simulation box, rather than the Rg of individual single chains.** Consequently, in a non-phase-separating system where chains are dispersed throughout the volume, the global Rg is high. Conversely, when the system undergoes phase separation into a compact droplet, the global Rg decreases significantly. This makes the global Rg an effective order parameter for monitoring the transition from a dispersed state to a condensed state, rather than a measure of internal chain expansion. We have made it clearer with a discussion with proper references in the revised manuscript as follows (**page 11, lines 224-229**):

"To monitor the transition from a dispersed to a condensed state, we additionally utilized the global radius of gyration (Rg) of all chains within the simulation volume as a collective order parameter. It is important to distinguish this system-wide measure from the single-chain Rg typically discussed in polymer scaling theories.[55-57] In our results, a decrease in global Rg signifies the formation of a compact multi-chain condensate."

We would further like to clarify that we have separately performed full-length FUS (FL-FUS, 526 residues) simulations at three salt concentrations in a bigger simulation box of $(40\ \text{nm})^3$ (shown in **Figure S6**) to

correlate the FL-FUS observations with those of the FUS-LCD. Nevertheless, to alleviate the Reviewer's concern we calculated the distribution of Rg of single chains inside the condensate as well as the global Rg of the full condensate to show that the single chain Rg is lower than the condensate Rg (see **Figure R2**). We have also included this figure in the SI (**Figure S10**).

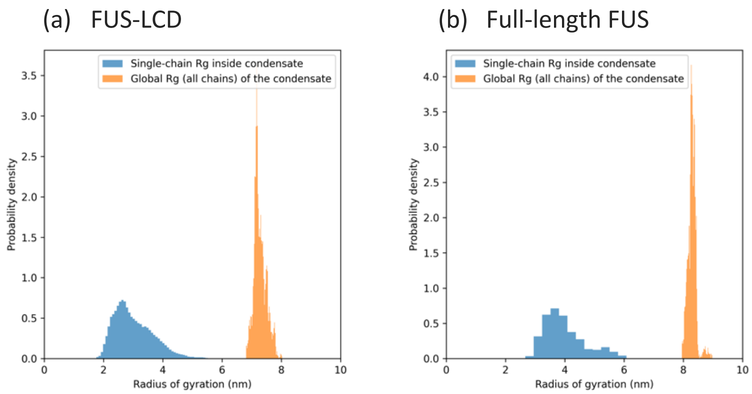

Figure R2. Distributions of single-chain Rg (blue) and global Rg (orange) for (a) 42 chain FUS-LCD condensate in (30 nm)^3 box and (b) 25 chain Full-length FUS condensate in (40 nm)^3 box, both simulated with 0.15M NaCl.

5. Followed by the above question, the authors should discuss the potential impact of the finite-size effects in this study or demonstrate that these effects do not affect the major conclusions.

This is an important point, not only in LLPS simulations but also for the simulation of other system undergoing phase-separation and phase transition. The finite size effect arises from the suppression of long-wavelength fluctuations inside the systems which get restricted by the small box size. However, MARTINI simulations benefit from the fact that the long-range interactions are not present. Both the Lennard-Jones and Coulomb interactions are cut-off at 1.1 nm and the rest is taken care of by a reaction field. The size of the boxes (30-40 nm cubes) is much higher than both the cut-off and the dimension of single chains (see **Figure R2**). Therefore, we do not expect to see artefacts from finite size effects. We have added the following text in the methods section (**page 8, lines 168-173**).

"While finite-size effects often arise from suppressed long-wavelength fluctuations during phase transitions, MARTINI simulations mitigate these issues by utilizing short-range interaction cutoffs and reaction fields. Given that our box dimensions (30 - 40 nm) are significantly larger than both the 1.1 nm cutoff and individual chain dimensions as well as the condensate dimensions (**Figure S10**), we do not expect significant finite-size artifacts."

6. Error bar should be added to the PMF curves in Fig. 2 and supporting data should be provided to show the convergence of the sampling.

We thank the Reviewer for this comment. We have now included error bars in Figure 2, also shown below as **Figure R3**:

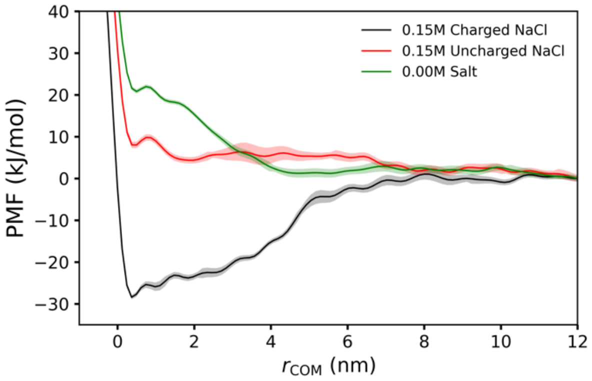

Figure R3. Effective attraction between FUS monomers through highly correlated medium of salt ions: Potential of mean force (PMF) between two FUS chains calculated by using well-tempered metadynamics. The two FUS chains are attractive in 0.15 M NaCl solution but become repulsive when the ionic charges are set to '0', that is, electrostatics are turned off; or when no excess ions are present at 0.0 M.

We have additionally provided the proof of convergence by plotting the metadynamics hill height against time. As this is a well-tempered metadynamics simulation, the hill height asymptotically approaches to zero when the simulation converges. This is included as **Figure S11**, also given below.

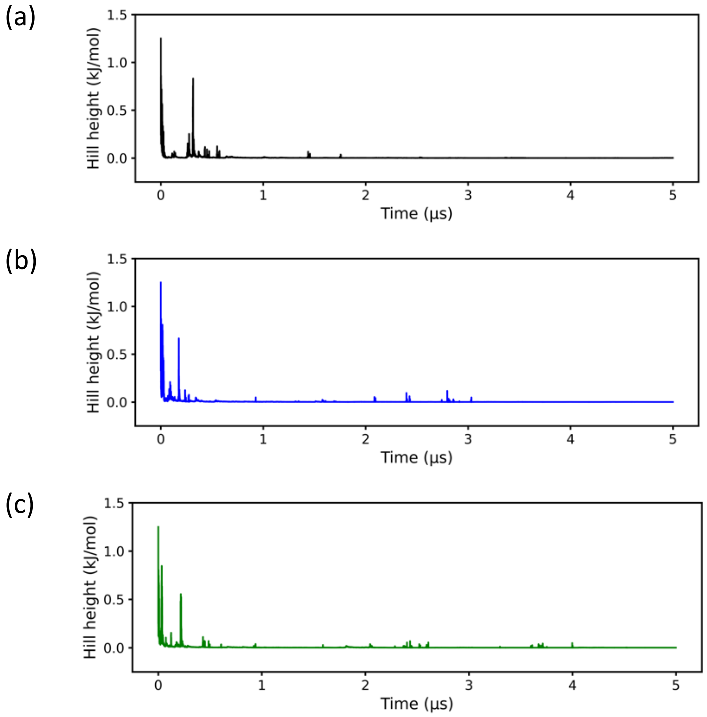

Figure R4. Proof of convergence of the well-tempered metadynamics simulations: metadynamics hill height against simulation time for (a) FUS-LCD in 0.15 M charged NaCl, (b) FUS-LCD in 0.15M uncharged NaCl, and (c) FUS-LCD in 0.0 M Salt.

7. Fig 3b shows that the artificial 'chargeless' FUS chains have phase separation at low salt concentration, and the authors attribute this to the interactions through highly correlated ionic plasma in Page 13. It seems that this is quite a general phenomenon, namely non-charged disordered proteins can undergo phase separation at low salt concentration. Can the authors have some discussions on the generalizability of this phenomenon? For example, does this phenomenon depend on the specific sequences? Additionally, how might the coarse-grained parameters for salts influence these observations?

Thank you for bringing up this interesting point. Yes, it is a general phenomenon for uncharged IDPs, and it indeed depends on the sequence features and intermolecular interactions. As already emphasized in the analytical theory, the protein-ion interactions should surpass the inter-protein interactions for this phenomenon to happen. A well-calibrated coarse-grained forcefield should capture these aspects.

8. The authors mentioned that "the PMF shows effective long-range attractive interactions that extend up to 12 nm length scale" in Page 26, this may be only due to the fact that the FUS protein is quite long and there are still some direct contacts when their COM distance is beyond than 12nm.

To ensure these interactions were not merely artifacts of direct contacts between the long FUS chains, we analyzed the correlation between the center-of-mass (COM) distance and the number of inter-monomeric contacts (**Figure R5**). We observed that direct residue-residue contacts reach zero at a COM separation more than ~5 nm, yet the PMF continues to show a favorable attractive gradient. This indicates that the long-range attraction is partially mediated by the ionic environment and solvent correlations, rather than being exclusively driven by direct physical contact between the protein chains.

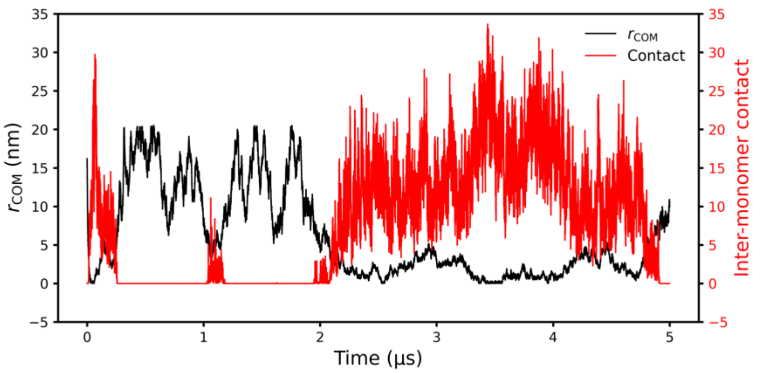

Figure R5. Trajectories of two collective variables, r_{COM} and inter-monomeric contact throughout the metadynamics trajectory for the FUS-LCD in 0.15 M NaCl solution.

9. Since analytical theory is used, is that possible to compare the enthalpy energies in the system before and after phase separation, as well as the entropy part? This will provide direct evidence for the driving force to the phase separation and support the major conclusions.

We appreciate this thoughtful comment from the reviewer. However plotting entropy and enthalpy of such a complex system might be not optimal as both quantities are combinations of contributions of

polymer, ions of both types and water (which is treated in the analytical theory implicitly via the incompressibility condition that excludes one component. Thus, integral entropy and enthalpy curves would be hard to interpret while focusing on a specific contribution, e.g. from the protein component could be misleading due to comparable contributions from the other components of the system. This is especially true of the Debye-Huckle component where both entropy and represent non-analytical power law dependencies of concentrations.

Reviewer #2

The manuscript combines computational (coarse-grained models in explicit solvent) and analytical methods at the mean-field level to describe the salt-dependent assembly and disassembly of FUS, an intrinsically disordered protein. The authors argue that at physiologically relevant salt concentrations, ion correlations can form long-range attractions (~10 nm), which in turn lead to the aggregation of FUS. At higher salt levels, the aggregate disassembles, and eventually, at excess salt concentration (>1 M), an aggregate formation (similar to that observed at lower salt levels) reappears. The manuscript focuses on a very important aspect of coacervate formation and convincingly combines analytical and computational tools to explain how electrostatics can control the phase segregation of charge-neutral molecules. In my opinion, this timely manuscript is suitable for publication after some minor revisions.

Thank you for your comments and for recommending the manuscript for publication after minor revisions. We have addressed all your comments in the revision. The pointwise replies are given below:

Major comments:

1. Line 99: The Voorn-Overbreek transition can be explained in the main text briefly

Thanks for the suggestion. We have added the following sentence on **page 5** (**lines 97-102**) of the revised manuscript:

"The classical VO approximation has been applied to treat coacervation of polyions by replacing short-range Flory-Huggins interaction term with electrostatic interactions between monomers of polyions in the DH approximation. In variation with the classical VO approximation here we consider short-range non-electrostatic interactions between monomers of the uncharged polymer and salt ions while treating interion interactions in the DH approximation."

2. I would assume that no long-range electrostatics were calculated in the simulations, which is reasonable given the high salt concentration. However, the manuscript can benefit from benchmarking regarding the cut-off distance (1.1 nm).
And
3. What is the underlying reason for choosing the dielectric constant 15?

(Comments #2 and #3 together): We thank the reviewer for these technical questions regarding the electrostatic treatment in our simulations. The choice of a 1.1 nm cut-off and a dielectric constant (ϵ) of 15 is specifically dictated by the parameterization of the MARTINI force field.

In the MARTINI framework, the non-bonded interactions are carefully calibrated as an integrated package; the Lennard-Jones and electrostatic potentials are tuned to work in tandem with a specific 1.1 nm cutoff.

Altering this distance without a comprehensive re-parameterization of the entire force field could disrupt the delicate balance of forces, potentially leading to unphysical results. This specific protocol has been extensively validated in recent literature for the study of FUS, TDP-43, and other IDPs, (Ingólfsson *et al. Biophysical Journal* **122**, 4370-4381 (2023); Zerze *et al*., *JCTC* **20**, 1646-1655 (2023); Thomasen *et al. JCTC* **18**, 2033-2041 (2022)) where it has consistently shown strong corroboration with experimental phase behavior.

Regarding the dielectric constant, the value of ϵ=15 is the standard setting for MARTINI simulations involving non-polar water models. Because the coarse-grained water beads represent four physical water molecules and lack an explicit dipole moment, the dielectric constant is set to 15 to account for the implicit screening and electronic polarization of the solvent environment. While we agree that a systematic benchmarking of these parameters, including the reaction field dielectric, would be an interesting endeavor for force field development, such an analysis is beyond the scope of the present study. Our goal is to apply these established and validated protocols to investigate the underlying physics of salt dependent reentrant LLPS, rather than to calibrate the underlying CG model itself.

4. What do the Authors think of the effect of the water dipole, which is, I believe, absent in Martini water models?

Simulations were performed using the Martini 3 coarse-grained force field. In this framework, water is modeled as a single non-polar interaction center. While this representation lacks an explicit dipole moment, the orientational polarizability and screening effects of the solvent are implicitly accounted for by using a relative dielectric constant of ϵ_r=15. This approximation is standard for Martini 3 and allows for the accurate reproduction of thermodynamic partitioning and protein-protein interaction strengths while maintaining the computational efficiency necessary to sample the long-timescale dynamics. However, there are further improvements of the MARTINI model by incorporating dipole (POL-MARTINI) or quadrupole (BMW-MARTINI) which can be used but at the same time increases the computational cost by 5-10 fold.

5. Line 416, the first sentence of the paragraph, appears to be inconsistent.

Thanks for pointing this out. We have rewritten the first part of the paragraph as (**page 23, lines 423-426**):

"We see from **Figure 5b** that at a low salt concentration regime, the condensate strongly absorbs ions. The effect is largely due to the Debye-Huckel (DH) correlation as the cancellation of the DH energy in the free energy functional shown in **Eq.**Error! Reference source not found. greatly diminishes due to absorption of ions in the condensate (blue curve)."

6. The ATP, which has a net charge and its own counterion (effectively a salt), has been identified as a hydrotropic molecule that affects the solvation properties of protein coacervates (Patel et al., Science, 2017). A recent study has shown that ATP can also achieve the same function, but on neutral polymers (Ayvaz et al., J. Phys. Chem. Lett., 2025). These experiments and simulations reported a non-monotonic

behavior on the conformation of neutral chains as a function of ATP. Can the Authors comment on the possible effect of their results on the hydrotropic properties of ATP?

We thank the reviewer for pointing out these highly relevant studies on the dual role of ATP as both a hydrotrope (Patel et al., 2017; DOI: 10.1126/science.aaf6846) and a modulator of neutral polymers (Ayvaz et al., 2025; DOI: 10.1021/acs.jpclett.5c02467).

While ATP is well-known as a biological hydrotrope that dissolves condensates at millimolar concentrations, Ayvaz et al. recently demonstrated that its effect on certain neutral macromolecules, specifically poly(N-isopropylacrylamide) or PNIPAM, is non-monotonic. In their study, low concentrations of ATP ([ATP]<0.15 M) promoted polymer aggregation via salting-out effects, whereas higher concentrations ([ATP] > 0.15 M) lead to dissolution. At [ATP] = 0 M, the polymers do not aggregate. While this non-monotonicity bears a superficial resemblance to the first re-entrant transition reported in our manuscript, the underlying physical driving forces are fundamentally different due to the distinct chemical compositions of the systems, as detailed below.

Firstly, the PNIPAM chains studied by Ayvaz et al. are characterized by purely hydrophobic side chains (Valine like), making the system essentially a neutral, hydrophobic polymer. In contrast, our system of interest (FUS-LCD) is primarily composed of polar amino acids (e.g., Serine and Tyrosine). Secondly, because PNIPAM is hydrophobic, it undergoes aggregation via a "salting-out" mechanism in the presence of ATP. Conversely, our polar system exhibits a "sucking-in" of ions at low-to-moderate concentrations, where the ionic medium creates long-range correlations that favor attraction between polar residues.

Given these fundamental differences the mechanisms driving their respective phase behaviors are qualitatively distinct and cannot be directly correlated. Nevertheless, we acknowledge the importance of these findings in the context of non-monotonic phase behavior and have added the following discussion to our conclusion (**page 26, lines 488-494**):

“Recent studies on neutral, hydrophobic polymers have shown that polyanions like ATP can induce non-monotonic phase behavior through salting-out effects at low concentrations followed by hydrotropic dissolution.[71] While this resembles the first re-entrant transition observed in our study, the mechanism we describe for polar uncharged IDPs like FUS-LCD is distinct. Unlike hydrophobic aggregation through ‘salting-out’, the phase separation at low ionic strength in FUS-LCD is driven by an 'ion-correlated' attraction where ions are partitioned into the condensate.”

Minor comments:

1. Snapshots in Figure 1 can be larger.

We have magnified the snapshots.

2. Line 230, the Authors still call the charge-neutral NA and Cl beads “salt”.

Thanks for pointing out. Changed.

3. Line 265/266, typos.

Thanks. The typos are corrected now.

4. Does Eq.2 miss the Boltzmann constant, given that T is the absolute temperature defined in the Methods section?

Thanks for the comment. The equations are constructed in reduced unit. Hence, k_B is equated to 1.

5. Figure 5b shows the salt concentration in the figure titles

Thanks. Corrected.

6. Figures 5d-f have a lower quality than other figures, and Figure 5f misses the right-aligned axis.

Thanks. We have improved the figure resolutions. In Figure 5f, both the black (low salt condition) and magenta (high salt conditions) lines correspond to ϕ_F^{Max}. Hence, the Y-axis on the right-hand side does not need labeling.

Reviewer #3

The work of Mondal & Shakhnovich is probably the most interesting paper on biomolecular condensates that I have read in a while and will be an essential contribution in the field, providing a clear and generalizable explanation for the salt dependent phase separation of uncharged or almost uncharged disordered proteins. The manuscript is thought provoking and provides not only a computational model but a theoretical framework that enables dissecting the various aspect involved in the studied re-entrant phase behavior. The results are particularly important because they also demonstrate the importance of properly accounting for ions in simulations, which is normally neglected in many of the typical coarse-grained simulations, potentially affecting interpretation of driving forces and even properties of the dense phase.

I don't have concerns but only few suggestions and curiosity that I help may improve the manuscript and perhaps the accessibility of results – I think that while the results are grounded in polymer physics, they are of essential importance for any scientist wanting to understand the interactions at play in biomolecular condensates.

I leave to the authors addressing any of my suggestion or curiosity, if they think they could improve the manuscript.

Thank you for the positive comments. We are glad that you found our work interesting. We have addressed all the queries below.

Suggestions/curiosity:

1. I think it would be helpful to define, if possible, a threshold in the $\Delta Q/N$ parameter to qualify phase separation vs small cluster formation. It would facilitate the interpretation of Figure 1 re-entrant phase diagram. I do agree the radius of gyration provides a strong qualifier, but it would add some sort of empirical boundaries that are helpful in the visualization of which phase occurs at which concentration.

We thank the reviewer for this suggestion. We agree that defining empirical boundaries for the $\Delta Q/N$ parameter would significantly improve the interpretability of the re-entrant phase diagram. As the reviewer correctly notes, while the radius of gyration (Rg) provides a strong indication of global condensation, $\Delta Q/N$ offers a complementary measure of the internal clustering state.

However, defining a single, universal threshold for $\Delta Q/N$ is non-trivial due to the inherent degeneracy of the parameter. Specifically, a system containing several small, dense clusters can yield a $\Delta Q/N$ value comparable to that of a single, large condensate, despite the different macroscopic phases. To resolve this, we rely on the combination of $\Delta Q/N$ (capturing local density) and Rg (capturing global spatial extent).

2. Line 227-228: “we note that these Rg… taken together … and do not refer to single chain Rg”. Can the author clarify this sentence? How are the Rg computed and what they do represent?

To quantify the transition between the dispersed and condensed phases, we calculated the global radius of gyration (Rg) of the entire 42-chain system. Computed as the root-mean-square distance of all beads from the system's total center of mass, this global Rg serves as a proxy for the spatial extent of the protein assembly. A high global Rg characterizes the dispersed phase where proteins occupy the available volume, while a sharp decrease in Rg signals the formation of a compact, phase-separated droplet. This collective measure is distinct from the single-chain Rg, which describes the conformational state of individual polymers within those phases. We have clarified it in the revised manuscript (**page 11, lines 224-229**).

3. Looking at the long-range effect of the potential of mean force between two FUS protein in Figure 2, I wonder whether the conformations of FUS molecules (e.g. the IDR region) are uniform or vary with salt concentration. It would be informative for future experimental attempts to provide simulated observables for single chains end-to-end and internal distances across the condensate to address if the effect of interaction is uniform across all FUS IDRs and whether it does impact equally the overall sequence or specific internal distances.

We thank the reviewer again for the suggestion, following which we have carried out end-to-end distance analysis for each chain present in the system and plotted the average value (with error bars) in **Figure R6** (below). The same is added to the Supporting Information as **Figure S12**.

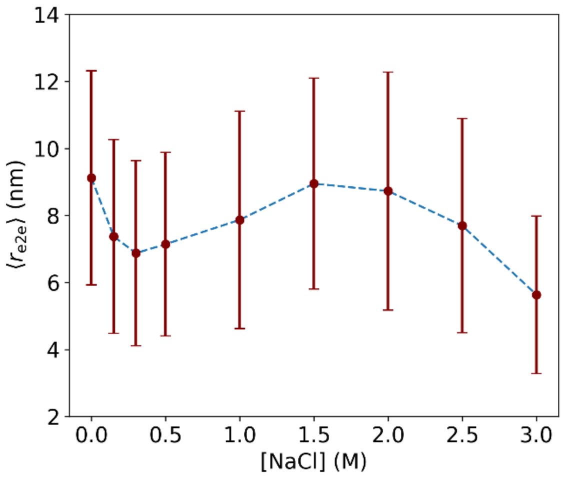

Figure R6. Average end-to-end distance, <r_{e2e}> of FUS-LCD chains in the system, against the NaCl concentration. <r_{e2e}> exhibits a non-monotonic behavior: it decreases when FUS-LCD chains undergo phase separation and increases when the condensate dissolves.

4. Line 265-266: two typos. Predcited instead of predicted.
and
5. Line 373. Eq: 4, typo (a square appears instead of a mathematical symbol)

(Reply for comments #4 and #5): Corrected. Thanks for pointing out.

6. Figure 5: panel b would benefit of a graphical highlighting that vertical dashed lines represent phi_I^OUT

Thanks for the suggestion. We have added the labels.

7. I would suggest promoting Fig S9 in the main text because of the importance in the comparison of systems behavior. I wonder if the authors could consider adding also some comments on phase separation of polyelectrolyte systems in high salt, as shown for nucleic acids – if it would operate according to a similar depletion mechanism.

We appreciate the reviewer's suggestion to promote Figure S9 to the main text. However, we have decided to keep it in the Supporting Information to maintain a clear narrative focus on the novel physics of uncharged IDPs, as bringing polyampholyte data into the main text might distract from our central discovery of ion-correlation-driven attraction.

Regarding polyelectrolytes and nucleic acids at high salt, our entropy-driven crowding mechanism may be relevant, but outcomes are highly sensitive to sequence features. As shown by Wessén *et al*. (Ref. 61), re-entrance in polyampholytes depends on the parameter γ (the ratio of volume change upon association to ionic correlation strength); only sequences with $\gamma < 2$ exhibit re-entry. Predicting outcomes for DNA-like polyanions is currently premature, as it depends on complex factors like complementary polycation chain, counterion valency, and hydration thermodynamics. Consequently, we have refrained from further comments pending future systematic studies.

8. This brings me to my personal curiosities that may be of interest to others to. Is the depletion effect at high molar salt true for all sequences? And is the disordered nature of the sequence relevant to it or would occur also for simply rigid domains? I think it would be important to explicit how generalizable is the system and what is the importance of being disordered or not.

The reviewer raises an important point regarding the generalizability of our observations. The observed phase behavior is sequence-dependent and is not a universal feature of all intrinsically disordered proteins (IDPs). The specific patterning of amino acid residues governs the interaction strength and the system's sensitivity to ionic screening. Consequently, IDPs with different sequence compositions will exhibit distinct phase boundaries and varying responses to high molar salt, as discussed in the previous reply.

Regarding the role of structural disorder, the conformational flexibility of IDPs is central to our current model. The disorder allows for a vast ensemble of states that can maximize favorable inter-chain contacts through fluctuation-induced attractions. While it is theoretically possible for structured proteins or rigid domains to exhibit similar salt-induced transitions (e.g., via the Hofmeister effect or specific surface-patch interactions), their fixed tertiary architectures likely impose different physical constraints.

Our current framework is highly *generalizable to nearly neutral IDPs with low net charge density*, where electrostatic correlations are the primary driver of phase separation. However, whether structured domains follow the same physics or require a modified treatment remains an open question. We agree that further investigation is necessary to delineate the boundaries between disordered and structured systems. In this context, and we have added the following sentences to the revised manuscript to discuss these limitations and the scope of our model (**page 29, lines 556-560**):

"These findings not only provide a microscopic understanding of the reentrant LLPS of FUS but also suggest a generalizable framework for other IDPs with low charge density. However, whether structured domains follow the same physics or require a modified treatment remains to be understood."

9. Can one think about polyampholyte charges (and perhaps polyelectrolyte) as disruptors of the ion correlations in the ion plasma?

We agree with the reviewer's insightful observation that polyampholytes and polyelectrolytes can be fundamentally conceptualized as disruptors of the ion plasma's equilibrium. In a bulk electrolyte, mobile ions form a statistically uniform plasma characterized by symmetric screening and well-defined correlation lengths. The introduction of a charged polymer, however, imposes a constrained, patterned geometry of charges that disrupts this symmetry. This fixed charge density modulates the local electrostatic landscape, compelling the surrounding ion atmosphere to reorganize and distort its correlation lengths to accommodate the polymer.

Furthermore, the conformational fluctuations inherent to these polymers generate a time-dependent electric field. This stochastic field can, in turn, modulate and propagate correlations throughout the plasma, potentially altering the long-range, correlation-induced attractions necessary for phase separation. We concur that systematic experimental and theoretical studies are required to fully quantify how these macromolecular perturbations dictate the macroscopic thermodynamics of the system. We thank the Referee for this perspective, which identifies a significant direction for future investigation.

10. In general, would additional components partitioning with FUS in the cell disrupt the ion plasma? I understand this goes beyond this work and may be an avenue for future works, but some speculation on the physiological context would be interesting.

While a definitive conclusion regarding the impact of additional cellular components on ion plasma disruption remains speculative, it is highly probable that the partitioning of RNAs and auxiliary proteins with FUS introduces significant charge heterogeneities that further modulate local electrostatic correlations. This multi-component environment may either amplify or buffer the ion-mediated forces driving phase separation, indeed representing an avenue for future research into how cellular physiology tunes the condensate landscape through local ionic regulation. We thank the Referee for this stimulating suggestion and have noted this as a key direction for subsequent investigation.

11. What is the length scale of correlations that one can expect due to the ion plasma? And could they impact not only partitioning, but also organization or material properties of the condensate?

The length scale of correlation is determined by the Debye length or Debye radius. The change in the Debye radius, by changing the ionic valency, temperature, concentration etc., can indeed affect the phase behavior.
